# CircRNF10-DHX15 interaction suppressed breast cancer progression by antagonizing DHX15-NF-κB p65 positive feedback loop

**DOI:** 10.1186/s11658-023-00448-7

**Published:** 2023-04-26

**Authors:** Wenfang Zheng, Xuehui Wang, Yunhe Yu, Changle Ji, Lin Fang

**Affiliations:** Department of Breast and Thyroid Surgery, Shanghai Tenth People’s Hospital, School of Medicine, Tongji University, 301 Yanchangzhong Road, Shanghai, 200072 People’s Republic of China

**Keywords:** circRNF10, DHX15, p65, Feedback, Breast cancer

## Abstract

**Background:**

Breast cancer (BC) is a common threat to women. The continuous activation of nuclear factor kappa B (NF-κB) signaling pathway contributes to the development of BC. This study aimed to investigate the role of a circular RNA (circRNF10) in BC progression and regulating NF-κB signaling pathway.

**Methods:**

Bioinformatics analysis, RT-qPCR, subcellular fractionation, FISH, RNase R treatment, and actinomycin D assay were used to explore the expression and characteristics of circRNF10 in BC. The biological functions of circRNF10 in BC were analyzed by MTT assay, colony formation assay, wound healing assay, and Transwell assay. RNA pulldown and RIP assay were used to identify the interaction between circRNF10 and DEAH (Asp-Glu-Ala-His) box helicase 15 (DHX15). The impact of circRNF10-DHX15 interaction on NF-κB signaling pathway was explored by western blot, IF, and co-IP. Furthermore, dual-luciferase reporter assay, ChIP, and EMSA were performed to assess the effect of NF-κB p65 on DHX15 transcription.

**Results:**

CircRNF10 was downregulated in BC, and lower expression of circRNF10 was related to poor prognosis of patients with BC. CircRNF10 inhibited the proliferation and migration of BC. Mechanically, circRNF10-DHX15 interaction sequestered DHX15 from NF-κB p65, thereby inhibiting the activation of NF-κB signaling pathway. On the other hand, NF-κB p65 enhanced *DHX15* transcription by binding to the promoter of *DHX15*. Altogether, circRNF10 impaired the DHX15-NF-κB p65 positive feedback loop and suppressed the progression of BC.

**Conclusion:**

CircRNF10-DHX15 interaction suppressed the DHX15-NF-κB p65 positive feedback loop, thereby inhibiting BC progression. These findings provide new insights in the continuous activation of NF-κB signaling pathway and raised potential therapeutic approach for BC treatment.

**Supplementary Information:**

The online version contains supplementary material available at 10.1186/s11658-023-00448-7.

## Background

Breast cancer (BC) is the most frequently diagnosed malignancy among women worldwide [1]. Although notable progress has been made in early diagnosis and therapeutic strategies, BC is still a serious threat to women due to its high morbidity and mortality [2]. Therefore, exploring the mechanism and novel therapeutic strategies of BC are crucial to improve the prognosis of patients with BC.

Circular RNA (circRNA) is a group of covalently closed single-stranded non-coding RNA generated by backsplicing [3]. The unique circular structure makes circRNAs superior in stability than other non-coding RNAs, which makes circRNAs applicable to tumor diagnosis and treatment [4]. Recent studies have demonstrated that circRNAs control the fate of tumor in various ways such as miRNA sponge [5–7], protein decoys [8, 9], and encoding peptides [10, 11]. An increasing body of evidence suggests that circRNAs have important biological functions in BC progression [12–14]. Previous studies have indicated that circRNF10 could act as the sponge of miR-934 and miR-942-5p [15, 16]. Whether it could interact with proteins is still unknown.

The nuclear factor kappa B (NF-κB) signaling pathway is essential in immune diseases and cancer [17]. Constitutive activation of NF-κB signaling pathway has been observed in multiple tumors [18, 19]. The heterodimers formed by p65 (encoded by *RELA*) and p50 are in charge of gene transcription when activating the canonical NF-κB signaling pathway, and the malignant phenotypes of tumor cells are closely related to activation of canonical NF-κB [20]. However, the reasons and the regulators of the constitutive activation of NF-κB signaling pathway are still elusive.

Here we reported that circRNF10 was downregulated in BC and it inhibited BC progression both in vitro and in vivo. The DHX15-p65 positive feedback loop caused the continuous activation of NF-kB signaling in BC, and circRNF10 acted as a suppressor of this loop by interacting with DHX15. This study might provide new insights in the mechanism of BC progression and raise potential therapeutic strategies for BC treatment.

## Methods

### Bioinformatic analysis

Datasets that contained circRNA expression in breast cancer and non-tumor tissues were searched in Gene Expression Omnibus (GEO, https://www.ncbi.nlm.nih.gov/geo/) database and GSE101123 was found. Differentially expressed circRNAs were analyzed by R studio and visualized in volcano plot and box plot. The putative binding sites of p65 and *DHX15* promoter were predicted on JASPAR (https://jaspar.genereg.net/) and PROMO (http://alggen.lsi.upc.es/cgi-bin/promo_v3/promo/promoinit.cgi?dirDB=TF_8.3#opennewwindow).

### Patients, samples, and clinical information

A total of 108 patients with breast cancer were involved in this study according to the following inclusion criteria: (1) patients newly diagnosed with BC confirmed by at least two experienced pathologists, (2) patients without any severe chronic diseases or malignancies, and (3) patients without any anti-tumor therapy before surgery. BC tissues and adjacent tissues were dissected and then snap-frozen in liquid nitrogen immediately after surgery. The clinical information of participants was collected. All patients had given their informed consent. This study was approved by the Ethic Committee of Shanghai Tenth People’s Hospital.

### Cell culture, transfection, and lentivirus infection

BC cell lines, including MDA-MB-231 (SCSP-5043), MCF-7 (SCSP-531), MDA-MB-468 (TCHu136), and BT549 (TCHu 93), non-tumorigenic breast epithelial cell line MCF-10A (SCSP-575), and HEK293T cells (SCSP-502) were obtained from the Chinese Academy of Sciences (Shanghai, China). The conditions for cell culture were described in a previous study [21]. All siRNAs and their negative control (si-NC) were synthesized by IBSbio (Shanghai, China) and the sequences were listed in Additional file 1. The plasmids for GFP-fused wild-type (WT), truncated DHX15 and site-mutated DHX15 [P327E, T421A, N422K, Y485E (GFP-DHX15-MUT)] were purchased from IBSbio (Shanghai, China). SiRNAs and plasmids were transfected by Lipo8000 reagent (Beyotime, Shanghai, China). The lentiviral vector pLV-circRNA-Hygro (HarO Life, Shanghai, China) and pCDH-MSCV-MCS-EF1-GFP-puro (IBSbio, Shanghai, China) were applied to construct overexpression plasmid of circRNF10 and DHX15, respectively. After lentiviral packaging using GMeasy Lentiviral Packaging Kit (Genomeditech, Shanghai, China) and cell infection, the cells were selected by 1 μg/mL puromycin (Beyotime, Shanghai, China) or 100 μg/mL hygromycin B (HarO Life, Shanghai, China), according to the antibiotic resistance.

### RNA extraction, reverse transcription (RT), polymerase chain reaction (PCR), Sanger sequencing, and real time quantitative PCR (RT-qPCR)

Total RNA was extracted using TRIzol (Invitrogen, Carlsbad, CA, USA), complying with the instructions. Reverse transcription (RT) was performed using HiScript III RT SuperMix kit (Vazyme, Nanjing, China). PCR was conducted using 2 × Hieff Robust PCR Master Mix (YEASEN, Shanghai, China). PCR products were separated by agarose gel electrophoresis and sequenced by Sanger sequencing. Reverse transcription quantitative polymerase chain reaction (RT-qPCR) was performed with Hieff qPCR SYBR Green Master Mix (YEASEN, Shanghai, China). For the internal control of circRNF10, *18S* was used, while *ACTB* was used as the internal control of *DHX15*, *RNF10*, and *RELA*. The relative expression of target genes was analyzed by the 2^−ΔΔCt^ method. All primers were listed in Additional file 1.

### RNase R treatment and actinomycin D assay

Total RNA extracted from BC cells were digested with Rnase R (Geneseed, Guangzhou, China) and then detected by RT-qPCR. As for actinomycin D assay, after incubation with 1 μg/mL actinomycin D (YEASEN, Shanghai, China) for 0 h, 8 h, 16 h, and 24 h, RNA was extracted from BC cells to determine the abundance of circRNF10 and linear *RNF10*.

### Subcellular fractionation

The Ambion PARIS Kit (Invitrogen, USA) was used for subcellular fractionation following the manufacturer’s instructions. *U6* and *GAPDH* were employed as nuclear control and cytoplasmic control, respectively.

### Fluorescent in situ hybridization (FISH)

The Cy3-labled probe of circRNF10 for FISH was synthesized by GenePharma (Shanghai, China) and the FISH was conducted using Ribo Fluorescent In Situ Hybridization Kit (RiboBio, Guangzhou, China). The sequence of the probe was as follows: 5’-GGCTACAAATGCGCTCCTAGATGAA-3’. For nucleus staining, 4′,6-Diamidino-2-Phenylindole (DAPI) was used. Images were captured by fluorescence microscope (Leica, Germany).

### MTT assay, colony formation assay, wound-healing assay, and Transwell assay

These biological function analyses were conducted as previous described [21].

### Protein extraction and western blotting

Total proteins were extracted using RIPA (Beyotime, Shanghai, China) with 1 mM PMSF (Beyotime, Shanghai, China). Nuclear and cytoplasmic proteins were extracted using Nuclear and Cytoplasmic Protein Extraction Kit (Beyotime, Shanghai, China). Western blotting was conducted following previous procedures [21]. Antibodies used for western blotting and their dilutions were as follows: anti-DHX15 (1:1000, sc-271686, Santa Cruz Biotechnology, USA), anti-GFP (1:1000, sc-9996, Santa Cruz Biotechnology, USA), anti-NF-κB p65 (sc-8008, Santa Cruz Biotechnology, USA), anti-p-NF-κB p65 Ser 536 (1:1000, sc-136548, Santa Cruz Biotechnology, USA), anti-β-Actin (1:1000, sc-47778, Santa Cruz Biotechnology, USA), anti-cyclin D1 (1:1000, 2978s, CST, USA), anti-Lamin A/C (1:1000, 10298-1-AP, proteintech, USA), Dylight 800-goat anti-rabbit IgG (1:2000, A23920, Abbkine, USA), and Dylight 800-goat anti-mouse IgG (1:2000, A23910, Abbkine, USA).

### RNA pulldown, silver staining, and RNA binding protein immunoprecipitation (RIP)

The probe of circRNF10 for RNA pulldown was as follows: 5’-GGCUACAAAUGCGCUCCUAGAUGAA-3’ (GenePharma, Shanghai, China). Proteins binding to circRNF10 were pulled down using RNA pulldown Kit (BersinBio, Guangzhou, China) and separated by SDS-PAGE electrophoresis. Silver staining was then conducted using Protein Silver Stain Kit (YEASEN, Shanghai, China). The differential protein band was identified by mass spectrometry (IBSbio, Shanghai, China). RIP was conducted using RNA Immunoprecipitation Kit (BersinBio, Guangzhou, China), followed by RT-PCR and agarose gel electrophoresis. Anti-DHX15 (sc-271686, Santa Cruz Biotechnology, USA) and anti-GFP (sc-9996, Santa Cruz Biotechnology, USA) were used for RIP.

### Immunofluorescence (IF)

After fixation, permeabilization and blocking, the cells were incubated with primary antibodies and followed by incubation with secondary antibodies. Nucleus were stained with DAPI. Images were captured under a fluorescence microscope (Leica, Germany). Antibodies applied in IF assay were as follows: anti-DHX15 (sc-271686, Santa Cruz Biotechnology, USA), anti-NF-κB p65 (sc-8008, Santa Cruz Biotechnology, USA), Alexa Fluor 488-goat anti-mouse IgG(H + L) (YEASEN, Shanghai, China), and Cy3-goat anti-mouse IgG(H + L) (YEASEN, Shanghai, China).

### Coimmunoprecipitation (co-IP)

Briefly, cells were lysed by RIPA (Beyotime, Shanghai, China) and the cell lysate was added with antibodies. After incubation at 4 °C overnight, Protein A/G PLUS Agarose (Santa Cruz Biotechnology, USA) was added into the mixture and incubated at 4 °C overnight. The proteins were eluted and analyzed by western blotting. Antibodies used for co-IP were listed as follows: anti-DHX15 (sc-271686, Santa Cruz Biotechnology, USA) and anti-NF-κB p65 (sc-8008, Santa Cruz Biotechnology, USA).

### Dual-luciferase reporter assay and Chromatin Immunoprecipitation (ChIP)

Wild-type (WT) and deletion (Del) reporter plasmids of putative p65-*DHX15* promoter binding sites (IBSbio, Shanghai, China), pRL-TK plasmid, and h*RELA* plasmid were applied in dual-luciferase reporter assay, which was conducted using Dual Luciferase Reporter Gene Assay Kit (Beyotime, Shanghai, China). ChIP assay was conducted using Chromatin Immunoprecipitation Kit (BersinBio, Guangzhou, China) according to the manufacturer’s instructions. Anti-NF-κB p65 (#8242, Cell Signaling Technology, USA) was used for ChIP assay.

### Electrophoretic mobility shift assay (EMSA)

The EMSA was performed using the chemiluminescence EMSA kit (Beyotime, Shanghai, China) following the manufacturer’s instructions. The probes corresponding to the putative binding site of p65 on *DHX15* promoter were synthesized by IBSbio (Shanghai, China) and the sequences were showed in Additional file 1. Briefly, nuclear extracts of MCF-7 cells was obtained using Nuclear and Cytoplasmic Protein Extraction Kit (Beyotime, Shanghai, China). For regular EMSA, 20 μg nuclear extracts were incubated with biotin-labeled probes. The unlabeled competitive probes (cold probe and mutant probe) were used to validate the binding specificity, and anti-NF-κB p65 (#8242, Cell Signaling Technology, USA) was further used to perform super-shift assay.

### Xenografts experiment and immunohistochemistry (IHC)

Four-week-old female BALB/c nude mice purchased from SLAC (Shanghai, China) were divided into two groups randomly (*n* = 5 for each group). A total of 2 × 10^6^ MDA-MB-231 cells stably expressing circRNF10 or vector were injected subcutaneously. All mice were sacrificed after 6 weeks and tumors were collected. The weight and volume of tumors were determined. For IHC assay, tumors were fixed, dehydrated, embedded, and sliced. The sections were then stained with anti-Ki-67 (GB121142, Servicebio, Wuhan, China), anti-DHX15 (sc-271686, Santa Cruz Biotechnology, USA), and anti-NF-κB p65 (sc-8008, Santa Cruz Biotechnology, USA). Representative images were taken using Leica Microsystems (Germany).

### Statistical analysis

Statistical analysis was conducted with GraphPad Prism 8 (GraphPad Software, USA) and SPSS Statistics 20 (IBM, USA). All data were presented as mean ± standard deviation (SD). Comparisons of circRNF10 expression between paired specimens were analyzed by Wilcoxon matched-pairs signed-rank test. Unpaired Student’s *t*-test was used for unpaired samples. The relationships between circRNF10 expression and clinicopathological information were evaluated by *χ*^2^ test. Kaplan–Meier plot and Log rank test were used to evaluate the effect of circRNF10 expression on progression-free survival (PFS). Two-way ANOVA was conducted to analyze the results of MTT assay. Correlation between *DHX15* and *RELA* was analyzed using Pearson’s correlation coefficient. A *P* value < 0.05 was considered statistically significant.

## Results

### The expression and characteristics of circRNF10 in BC

**Fig. 1:**
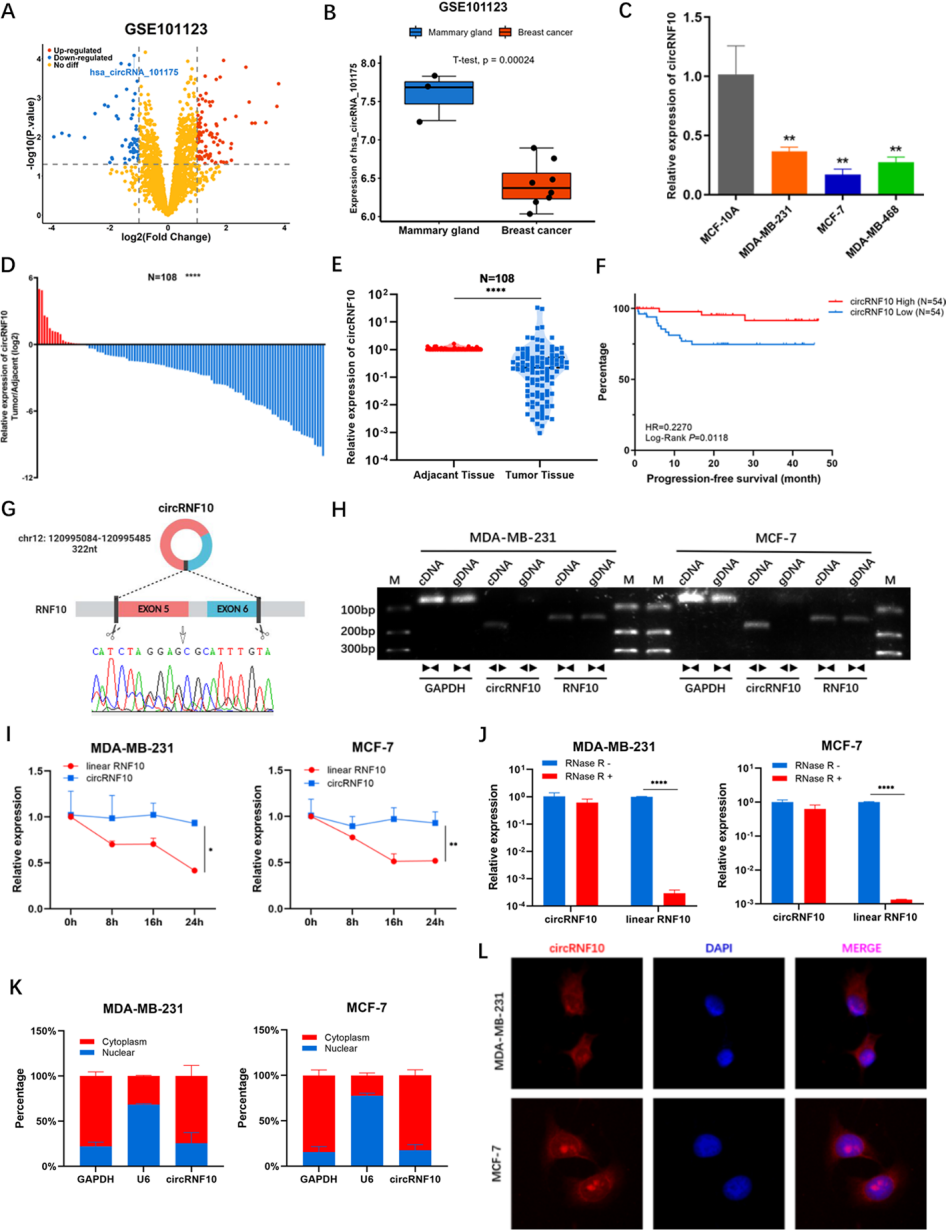
The expression and characteristics of circRNF10 in BC. **A** Volcano plot showing the differentially expressed circRNAs in BC in GSE101123 dataset. The cutoff was set at |log_2_(fold change)| > 1 and *P* < 0.05.** B** Box plot showing the expression of circRNF10 in mammary gland and BC according to GSE101123 dataset. **C** RT-qPCR analysis of circRNF10 expression in BC cells compared with MCF-10A cells. **D** RT-qPCR analysis of circRNF10 expression in BC tissues presented as the ratio of tumor/adjacent (*N* = 108). **E** RT-qPCR analysis of circRNF10 expression in BC tissues and adjacent tissues (*N* = 108). **F** Kaplan-Meier (K–M) plot presenting PFS of high and low circRNF10 expression group. **G** The formation of circRNF10 confirmed by Sanger sequencing. **H** RT-PCR analysis of circRNF10 and linear *RNF10* with divergent and convergent primers in MDA-MB-231 and MCF-7 cells. **I** The expression of circRNF10 after actinomycin D treatment in MDA-MB-231 and MCF-7 cells. **J** The expression of circRNF10 after RNase R treatment in MDA-MB-231 and MCF-7 cells. **K** Subcellular fractionation analysis of circRNF10, with U6 and GAPDH as enteral control of the nucleus and cytoplasm. **L** FISH analysis of circRNF10 localization in MDA-MB-231 and MCF-7 cells. Red: circRNF10, blue: DAPI. Error bars represent the means ± SD. *P < 0.05, **P < 0.01, ***P < 0.001, ****P < 0.0001

By analyzing differentially expressed circRNAs in BC using data in GSE101123, we discovered that circRNF10 (i.e., hsa_circRNA_101175) expression was significantly downregulated in BC tissue (Fig. 1A, B). We next validate the expression of circRNF10 in BC cells and tissues. The results suggested that circRNF10 was significantly downregulated in BC cell lines compared with MCF-10A cells (Fig. 1C). On the other hand, the expression of circRNF10 in malignant tissues was significantly lower than that in adjacent tissues from patients with BC (*N* = 108) (Fig. 1D, E). To explore the relationship between circRNF10 expression and the clinical characteristics of patients with BC, we divided patients with BC into high- and low-expression groups according to the median value of circRNF10 expression. As showed in Table 1, circRNF10 expression was negatively correlated to tumor stage, lymph node status, and recurrence/distant metastasis, while there were no significant associations between circRNF10 expression and patients’ age at diagnosis or molecular subtype. Moreover, analysis of PFS in each group indicated that lower circRNF10 expression led to shorter PFS (Fig. 1F). These results indicated that depleted circRNF10 might play a role in BC progression.

**Table 1:** The relationships between circRNF10 expression and clinical characteristics in patients with BC

Clinical characteristics	Total	circRNF10 expression		*P* value
		High (*N* = 54)	Low (*N* = 54)	
*Age at diagnosis*				
< 60	35	14	21	0.15
≥ 60	73	40	33	
*Molecular subtype*				
Luminal A	30	14	16	0.420
Luminal B	40	17	23	
HER2-positive	14	8	6	
TNBC	24	15	9	
*Stage*				
I and II	94	51	43	0.022^*^
III and IV	14	3	11	
*Lymph node status*				
Negative	81	45	36	0.046^*^
Positive	27	9	18	
*Recurrence or distant metastasis*				
No	93	51	42	0.012^*^
Yes	15	3	12	

^*^P < 0.05

The characteristics of circRNF10 were then explored. Sanger sequencing demonstrated that circRNF10 was generated from ring finger protein 10 (*RNF10*) by backsplicing exon 5 and exon 6 (Fig. 1G). Divergent primers could only amplify circRNF10 from cDNA rather than genomic DNA (Fig. 1H), which further validated the existence and circular structure of circRNF10. The half-life of circRNF10 was longer than linear *RNF10* in BC cells when treated with actinomycin D (Fig.1I). Additionally, circRNF10 resisted to RNase R digestion (Fig. 1J), showing that circRNF10 was more stable than the linear *RNF10*. Subcellular fractionation showed that circRNF10 was predominantly enriched in cytoplasm of BC cells (Fig. 1K). The results of FISH assay were consistent with nucleus–cytoplasmic separation (Fig. 1L). These results suggested that circRNF10 was a cytoplasmic circRNA generated by backsplicing.

Altogether, circRNF10 was depleted in BC and was associated with better prognosis of patients with BC.

### CircRNF10 acted as a tumor suppressor in vitro

**Fig. 2:**
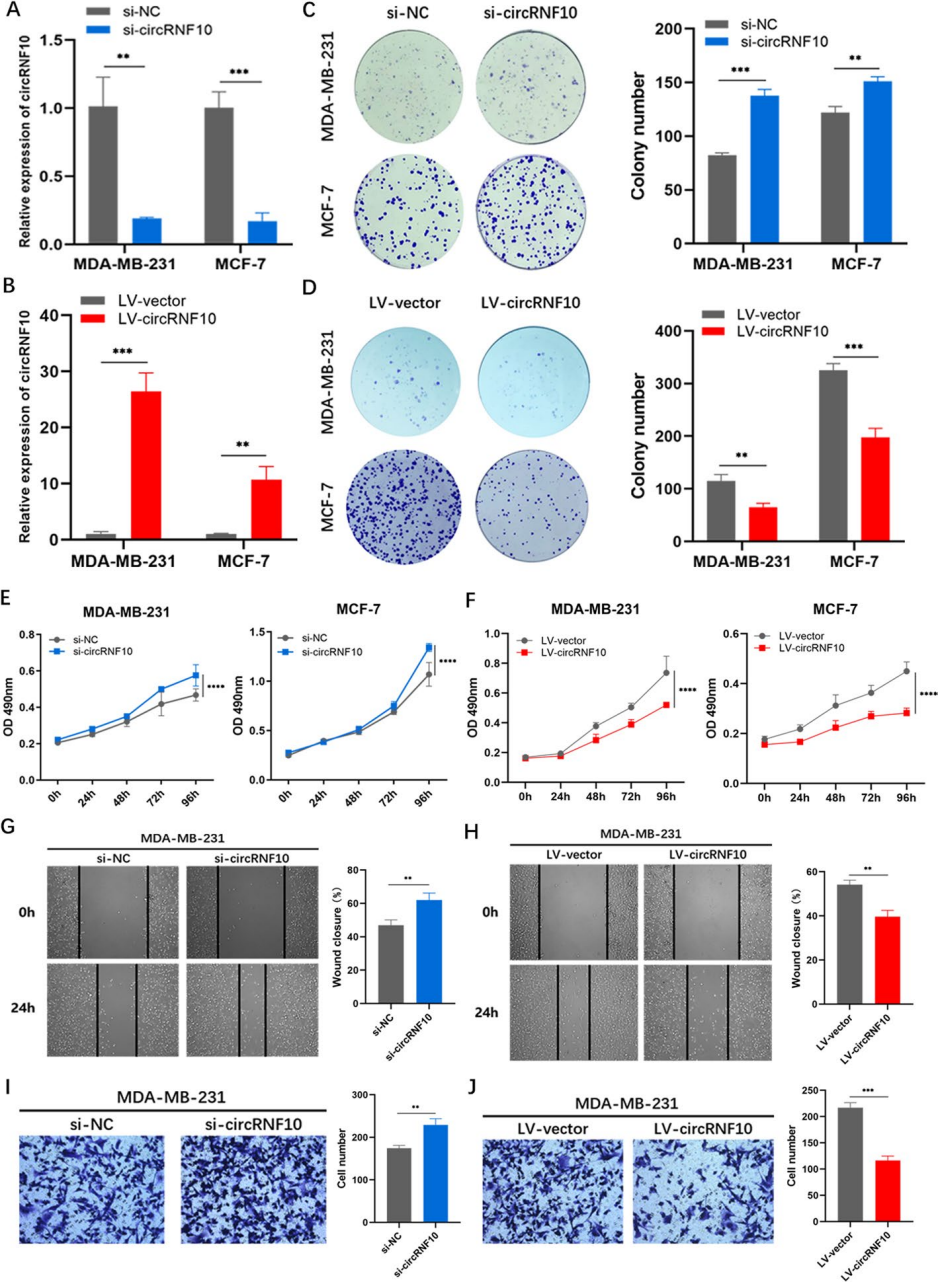
CircRNF10 was a tumor suppressor in BC cells. **A** RT-qPCR analysis of si-circRNF10 efficiency in BC cells. **B** RT-qPCR analysis of lentivirus (LV)-circRNF10 efficiency in BC cells. **C** Colony formation assay performed in si-NC and si-circRNF10 groups to evaluate the effect of inhibiting circRNF10 in BC cells (left) and the statistics of colony numbers (right). **D** Colony formation assay performed in LV-vector and LV-circRNF10 groups to evaluate the effect of overexpressing circRNF10 in BC cells (left) and the statistics of colony numbers (right). **E** MTT assay showing the proliferation of BC cells in si-NC and si-circRNF10 groups. **F** MTT assay showing the proliferation of BC cells in LV-vector and LV-circRNF10 groups. **G** Wound-healing assay comparing the migration of MDA-MB-231 cells in si-circRNF10 group with control (left) and the statistics of wound closure percentage (right). **H** Wound-healing assay comparing the migration of MDA-MB-231 cells in LV-circRNF10 group with control (left) and the statistics of wound closure percentage (right). **I** Transwell assay showing the migration of MDA-MB-231 cells transfected with si-NC or si-circRNF10 (left) and the number of migrated cells (right). **J** Transwell assay showing the migration of MDA-MB-231 cells expressing LV-vector or LV-circRNF10 (left) and the number of migrated cells (right). Error bars represent the means± SD. *P < 0.05, **P < 0.01, ***P < 0.001, ****P < 0.0001

To investigate the biological functions of circRNF10 in BC, siRNAs (si-circRNF10, circRNF10 si-2, and circRNF10 si-3) were used to knockdown circRNF10 expression (Fig. 2A and Additional file 2: Fig. S1A). Lentivirus was used to establish BC cell lines that stably overexpressed circRNF10 (Fig. 2B). Subsequent biological function analyses suggested that knocking down of circRNF10 promoted cell viability, proliferation, clonogenicity, and migration of BC cells, while overexpressing circRNF10 showed the contrary effects (Fig. 2C–J and Additional file 2: Fig. S1B–D). Considering that circRNF10 si-2 and si-3 had minor interference on circRNF10 expression or malignant phenotypes of BC cells, only si-circRNF10 was used in the following experiments (Additional file 2: Fig. S1A–D). These results demonstrated that circRNF10 inhibited tumor proliferation and migration of BC in vitro.

### CircRNF10 interacted with the oncogenic protein DHX15 in cytoplasm of BC cells

**Fig. 3:**
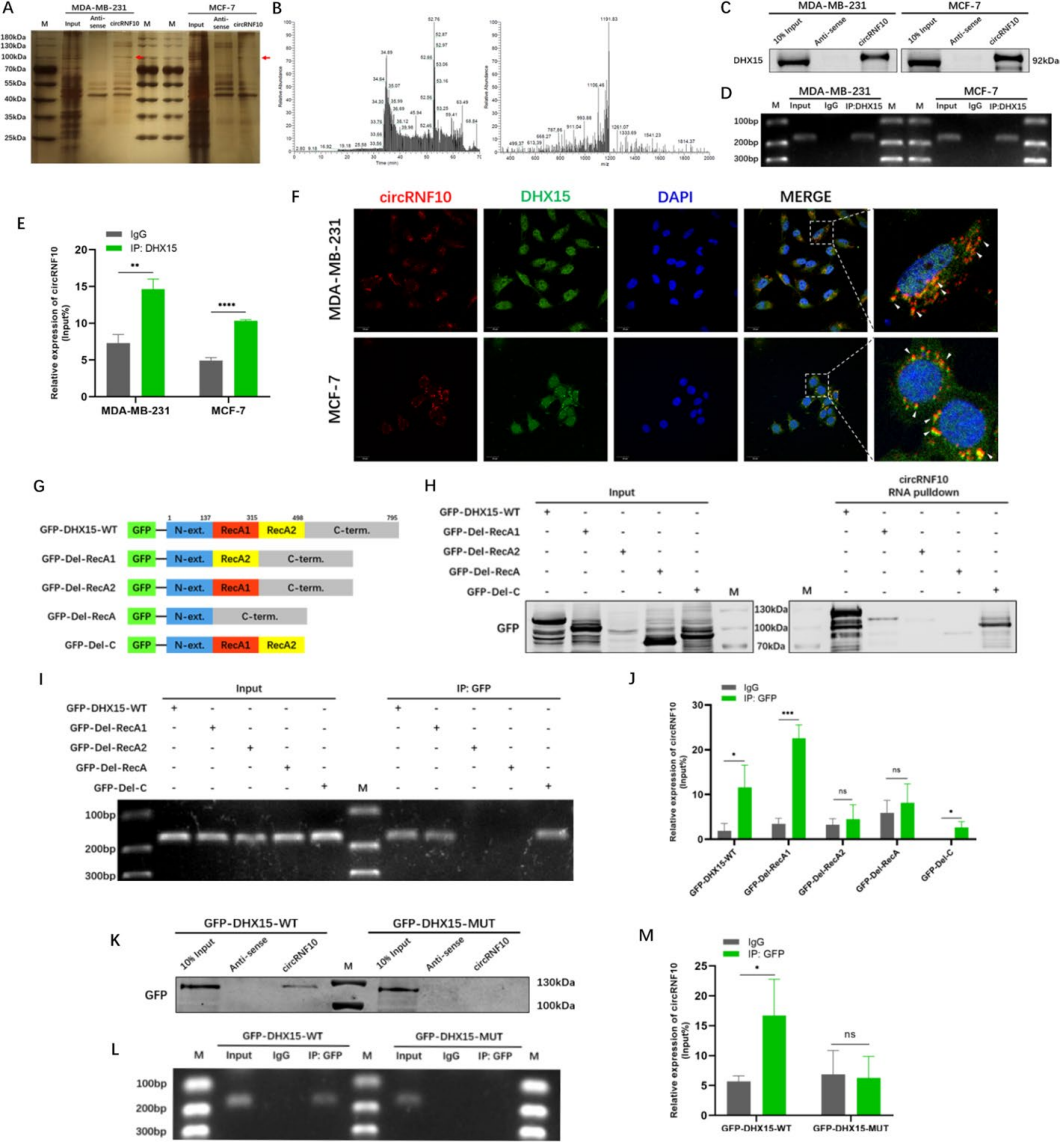
CircRNF10 interacted with DHX15 in the cytoplasm of BC cells. **A** Silver staining of the products of RNA pulldown in BC cells by the probe of circRNF10. Red arrow: differential protein band. **B** Mass spectrometry results of the differential protein band. **C** Western blotting analysis of the products of RNA pulldown. **D** RT-PCR analysis of the products of RIP assay in BC cells. **E** RT-qPCR analysis of the products of RIP assay. **F** FISH combined with IF to detect the colocalization of circRNF10 and DHX15 in BC cells. Red: circRNF10, green: DHX15, blue: DAPI. White arrows marked the colocalization of circRNF10 and DHX15. **G** Schematic illustration of the GFP-labeled truncations of DHX15. N-ext: N-extension of DHX15, C-term: C-terminal of DHX15. **H** Western blotting analysis of the products of RNA pulldown in HEK293T cells, which expressed exogenous DHX15 truncations. **I** RT-PCR analysis of the products of RIP assay in HEK293T cells that expressed exogenous DHX15 truncations.** J** RT-qPCR analysis of the products of RIP assay in HEK293T cells that expressed exogenous DHX15 truncations. **K** Western blotting analysis of the products of RNA pulldown in HEK293T cells that expressed GFP-DHX15-MUT (P327E, T421A, N422K, and Y485E). **L** RT-PCR analysis of the products of RIP assay in HEK293T cells that expressed GFP-DHX15-MUT (P327E, T421A, N422K, and Y485E). **M** RT-qPCR analysis of the products of RIP assay in HEK293T cells that expressed GFP-DHX15-MUT (P327E, T421A, N422K, and Y485E). Error bars represent the mean ± SD. *P < 0.05, **P < 0.01, ***P < 0.001, ****P < 0.0001, ns: no significance

The regulatory mechanisms of circRNAs were closely related to the subcellular localization of circRNAs [3]. Considering that circRNF10 was predominantly localized in the cytoplasm of BC cells, we assumed that circRNF10 regulated BC progression by interacting with RNA binding protein (RBP). Therefore, we designed the probe of circRNF10 and conducted RNA pulldown. Silver staining showed that there was a differential protein band near 100 kDa in the RNA pulldown products (Fig. 3A). The following mass spectrometry identified that the differential protein was most likely DEAH (Asp-Glu-Ala-His) box helicase 15 (DHX15) (Fig. 3B). The interaction between circRNF10 and DHX15 was further validated by western blotting analysis of RNA pulldown products (Fig. 3C) and RIP (Fig. 3D, E). In addition, FISH combined with IF indicated that circRNF10 and DHX15 were colocalized in the cytoplasm of MDA-MB-231 and MCF-7 cells, which further confirmed the circRNF10-DHX15 interaction in the cytoplasm (Fig. 3F). DHX15 includes two RNA binding domains (RecA1 and RecA2), and the C-terminal domain (CTD) of DHX15 is in charge of protein interaction, which regulates RNA binding [22]. To identify the domain of DHX15 that circRNF10 interact with, we first constructed the GFP-labeled truncations of DHX15 (Fig. 3G). The following RNA pulldown and RIP assay suggested that the interaction between circRNF10 and DHX15 was weakened when RecA2 domain was truncated, which indicated that circRNF10 mainly binds to RecA2 domain of DHX15 (Fig. 3H–J). Previous studies had indicated that the residues P327, T421, N422, and Y485 in RecA2 domain were in charge of RNA binding [22, 23]. We noticed that the interaction between circRNF10 and DHX15 vanished when these residues were mutated [P327E, T421A, N422K, and Y485E (GFP-DHX15-MUT)] (Fig. 3K–M). The results demonstrated that the circRNF10-DHX15 interaction was dependent on these key residues in RecA2 domain of DHX15.

DHX15 was reported to promote BC progression in previous studies [24, 25]. Consistently, our study demonstrated that the upregulated DHX15 in BC was related to the poor prognosis of patients with BC (Additional file 2: Fig S2A–D), and the functional analyses of DHX15 suggested that downregulation of DHX15 led to decreased proliferation and migration of BC cells, while overexpression of DHX15 showed opposite effects on BC progression (Additional file 2: Fig S2E–P). Altogether, circRNF10 interacted with the oncogenic DHX15 in the cytoplasm of BC cells.

### CircRNF10 suppressed the NF-κB signaling pathway by circRNF10-DHX15 interaction

**Fig. 4:**
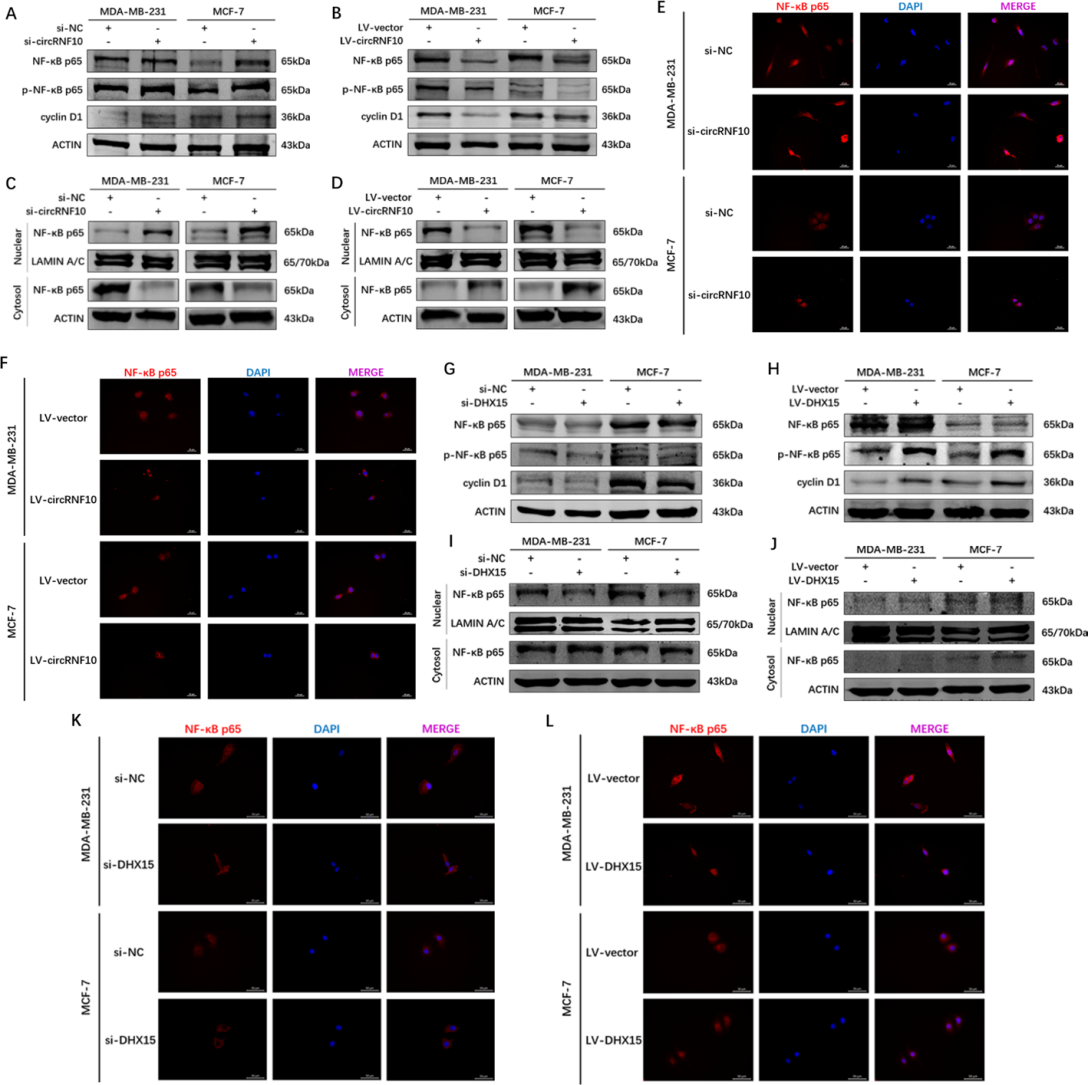
CircRNF10 and DHX15 were involved in the activation of NF-κB signaling pathway. **A** Western blotting assay showing the expression of p65, p-p65 (S536), and cyclin D1 in BC cells in si-circRNF10 group and control. **B** Western blotting assay showing the expression of p65, p-p65 (S536), and cyclin D1 in BC cells in LV-circRNF10 group and control. **C** Western blotting presenting the cytoplasmic and nuclear p65 levels in si-NC group and si-circRNF10 group of BC cells. **D** Western blotting presenting the cytoplasmic and nuclear p65 levels in LV-vector group and LV-circRNF10 group of BC cells. **E** IF assay indicating the subcellular localization of p65 in si-NC group and si-circRNF10 group of BC cells. Red: p65, blue: DAPI.** F** IF assay indicating the subcellular localization of p65 in LV-vector group and LV-circRNF10 group of BC cells. Red: p65, blue: DAPI. **G** Western blotting analysis showing the influence of si-DHX15 on expression of p65, p-p65 (S536), and cyclin D1 in BC cells. **H** Western blotting analysis showing the influence of LV-DHX15 on expression of p65, p-p65 (S536), and cyclin D1 in BC cells. **I** Western blotting analysis presenting the cytoplasmic and nuclear p65 levels in si-NC group and si-DHX15 group of BC cells. **J** Western blotting analysis presenting the cytoplasmic and nuclear p65 levels in LV-vector group and LV-DHX15 group of BC cells. **K** IF assay showing the subcellular localization of p65 in si-NC group and si-DHX15 group of BC cells. Red: p65, blue: DAPI. **L** IF assay showing the subcellular localization of p65 in LV-vector group and LV-DHX15 group of BC cells. Red: p65, blue: DAPI

Previous studies had indicated that DHX15 was a regulator of the NF-κB signaling pathway [26, 27], thus we hypothesized that the circRNF10-DHX15 interaction was involved in the regulation of NF-κB signaling pathway in BC. To validate this hypothesis, the impacts of circRNF10 and DHX15 on NF-κB signaling pathway were first explored. The result showed that circRNF10 inhibited the phosphorylation of p65 (S536) and the expression of cyclin D1 (Fig. 4A, B). In addition, subcellular fractionation and IF assay indicated that circRNF10 suppressed the nuclear translocation of p65 in BC cells (Fig. 4C–F). On the contrary, DHX15 promoted the expression of p-p65 (S536) and cyclin D1 in BC cells (Fig. 4G, H), thereby activating the nuclear translocation of p65 (Fig. 4I–L). Together, these results suggested that both circRNF10 and DHX15 were involved in the regulation of the NF-κB signaling pathway.

**Fig. 5:**
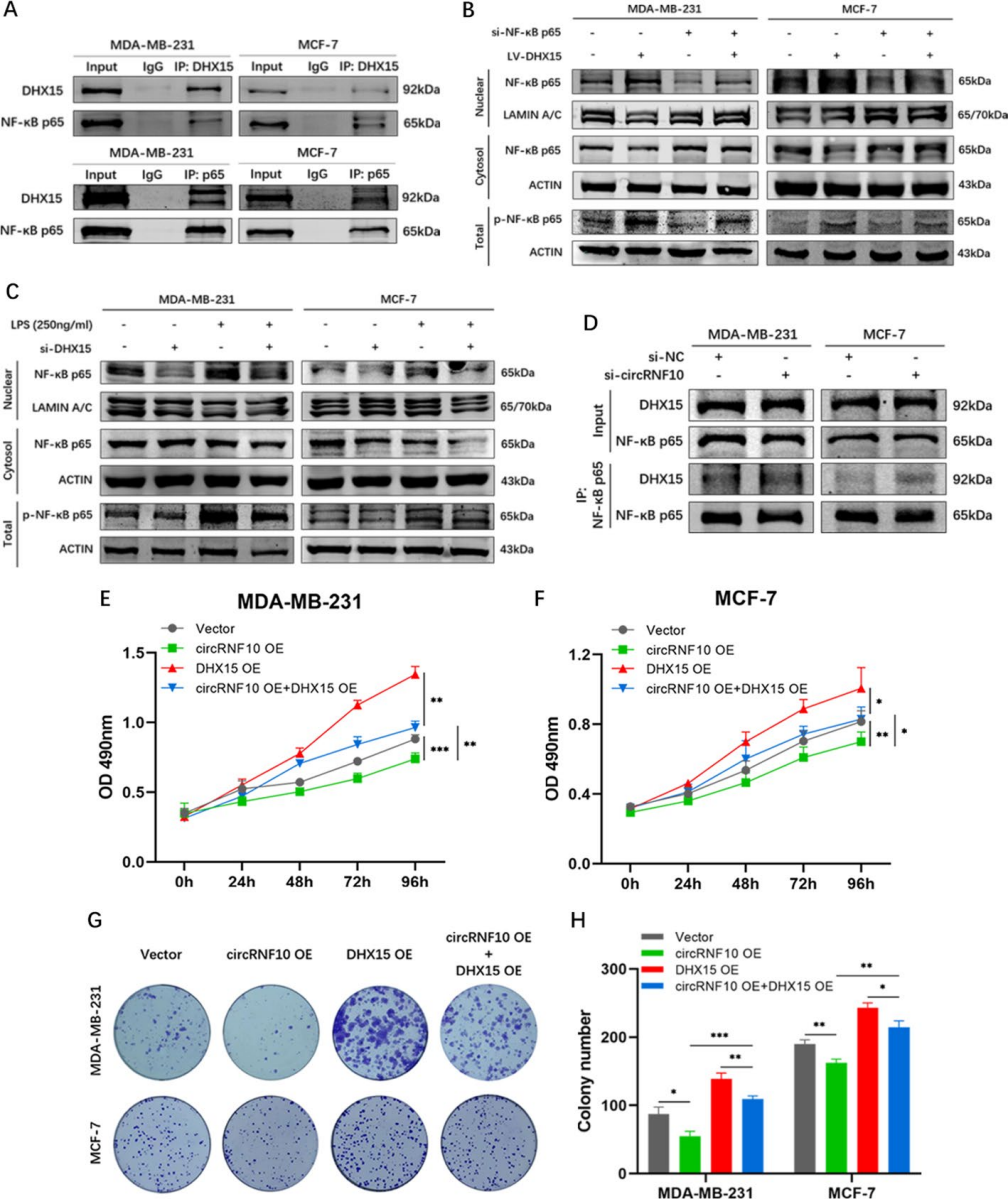
CircRNF10 inhibited BC progression by antagonizing DHX15-p65 interaction. **A** Co-IP assay detecting the interaction between endogenous DHX15 and p65 in BC cells. **B** Western blotting analysis presenting the influence of LV-DHX15 on the p-p65 (S536) levels in total protein and the cytoplasmic and nuclear p65 levels, when p65 was suppressed in BC cells. **C** Western blotting analysis presenting the influence of si-DHX15 on the p-p65 (S536) levels in total protein, and the cytoplasmic and nuclear p65 levels when BC cells were treated with LPS (250 ng/ml). **D** Co-IP assay presenting the change of DHX15-p65 interaction after inhibiting circRNF10 in BC cells. **E** MTT assay showing the influence of circRNF10 overexpression on the proliferation of DHX15 overexpressed MDA-MB-231 cells. **F** MTT assay showing the influence of circRNF10 overexpression on the proliferation of DHX15 overexpressed MCF-7 cells. **G** Colony formation assay showing the effect of circRNF10 overexpression on the proliferation of DHX15 overexpressed BC cells. **H** The number of colonies. Error bars represent the mean ± SD. *P < 0.05, **P < 0.01, ***P < 0.001, ****P < 0.0001

Although previous studies had indicated that the NF-κB signaling pathway could be activated by DHX15 [27], how it was activated is still elusive. Here we performed a co-IP assay and the result indicated that DHX15 interacted with p65 in BC cells (Fig. 5A). Further rescue experiments showed that overexpression of DHX15 did not enhance p65 nuclear translocation when p65 was inhibited (Fig. 5B and Additional file 2: Fig. S3A, B), while lipopolysaccharide (LPS)-induced nuclear translocation of p65 was significantly rescued by downregulation of DHX15 in BC cells (Fig. 5C and Additional file 2: Fig. S3C, D). These results indicated that DHX15-p65 interaction was required in the activation of the NF-κB signaling pathway in BC.

Considering both circRNF10 and p65 could interact with DHX15, whether there was a competition between these interactions was unclear. The co-IP assay exhibited that the DHX15-p65 interaction increased after inhibition of circRNF10 (Fig. 5D), demonstrating that circRNF10 competitively impaired the interaction between DHX15 and p65 via circRNF10-DHX15 interaction in BC. Accordingly, the rescue experiments were performed to validate that circRNF10 played its suppressive role in BC via binding to DHX15. Compared with overexpressing DHX15 alone, circRNF10 could partially eliminate the tumor-promoting effects induced by elevated DHX15 expression (Fig. 5E–H). Furthermore, the tumor-suppressive role of circRNF10 on BC proliferation was partially abolished by knocking down of DHX15 (Additional file 2: Fig S4A–D). Collectively, these findings verified that circRNF10 inhibited BC progression and suppressed the NF-κB signaling pathway by circRNF10-DHX15 interaction.

### DHX15-p65 positive feedback loop in BC was antagonized by circRNF10

**Fig. 6:**
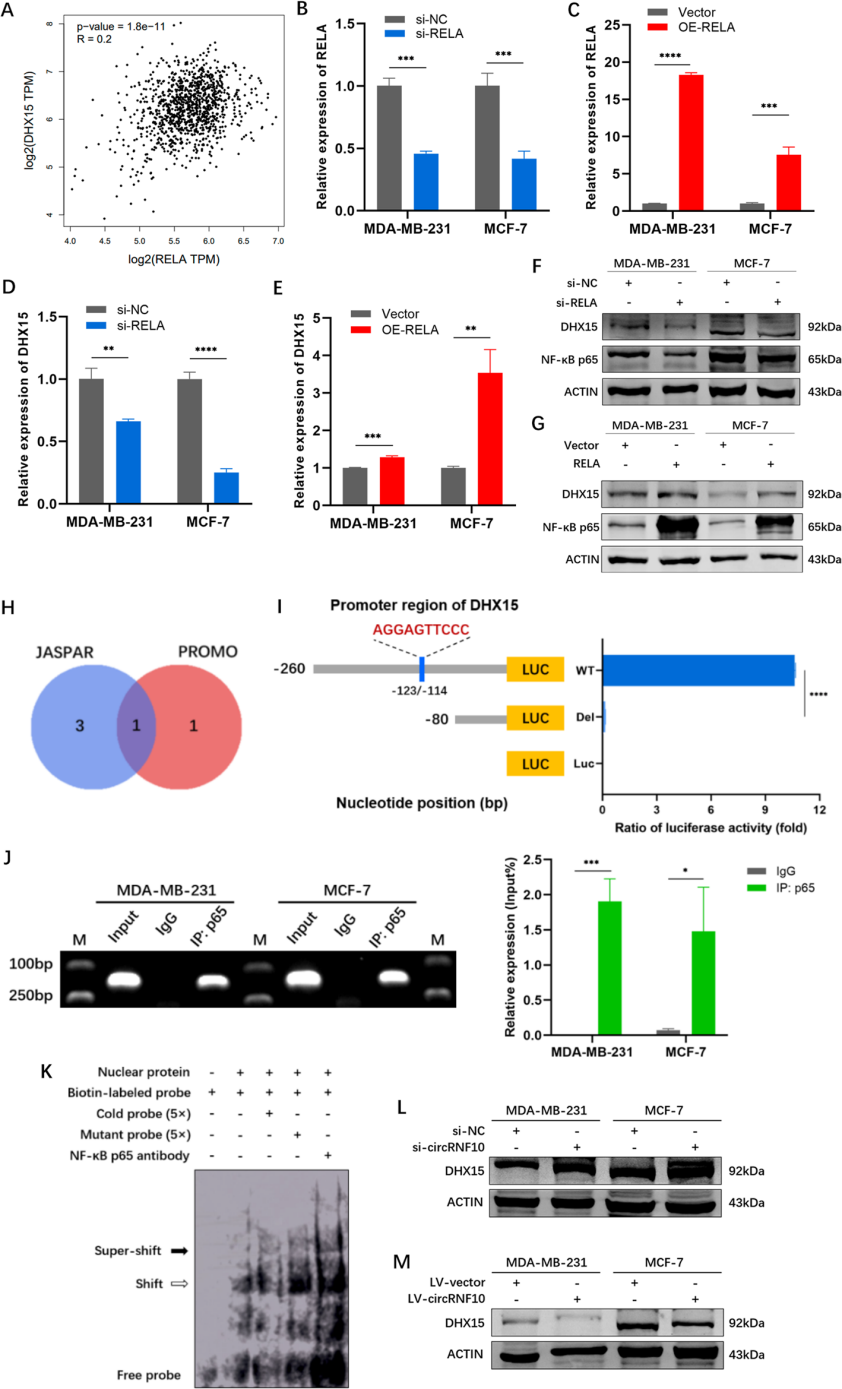
DHX15-p65 positive feedback loop in BC was impaired by circRNF10. **A** Correlation between *DHX15* and *RELA* expression in BC according to the GEPIA2 database. **B** RT-qPCR analysis showing the efficiency of si-*RELA* in BC cells. **C** RT-qPCR analysis showing the efficiency of OE-*RELA* in BC cells. **D** RT-qPCR analysis showing the expression of *DHX15* after suppressing *RELA*. **E** RT-qPCR analysis showing the expression of *DHX15* after overexpressing *RELA*. **F** Western blotting assay presenting the levels of DHX15 and p65 in si-NC and si-*RELA* groups of BC cells. **G** Western blotting assay presenting the levels of DHX15 and p65 in vector and OE-*RELA* groups of BC cells. **H** Schematic illustration showing the predicting of p65 binding sites on *DHX15* promoter region. **I** Schematic illustration showing the reporter plasmids cloning *DHX15* promoter region with different length (left) and dual-luciferase assay detecting the binding of p65 and *DHX15* promoter region (right). **J** ChIP assay showing that p65 directly binds to the promoter region of *DHX15* by agarose gel electrophoresis (left) and qPCR (right). **K** EMSA suggesting that the complexity of p65 and *DHX15* promoter existed. **L** Western blotting assay showing the expression of DHX15 after inhibiting circRNF10 in BC cells. **M** Western blotting assay showing the expression of DHX15 after overexpressing circRNF10 in BC cells. Error bars represent the mean ± SD. *P < 0.05, **P < 0.01, ***P < 0.001, ****P < 0.0001

NF-κB p65 is a transcription factor that promotes the transcription of its target genes by binding to their promoters [28]. Interestingly, we noticed that the expression of *DHX15* was positively related to *RELA* in BC (Fig. 6A). Therefore, we assumed that p65 could promote transcription of *DHX15*. Si-*RELA* and OE-*RELA* were transfected into BC cells, respectively (Fig. 6B, C). RT-qPCR showed that p65 was an activator of *DHX15* transcription (Fig. 6D, E). The change of protein level of DHX15 was consistent with the alteration of transcription (Fig. 6F, G). To explore whether p65 could bind to the promoter region of *DHX15*, the p65 binding sites on *DHX15* promoter region were predicted using JASPAR (https://jaspar.genereg.net/) and PROMO database (http://alggen.lsi.upc.es/cgi-bin/promo_v3/promo/promoinit.cgi?dirDB=TF_8.3#opennewwindow), and a putative binding site (−123 to −114) was found (Fig. 6H, I). The luciferase activity was significantly reduced when the putative binding site was truncated in Del group (Fig. 6I), indicating that the p65 promoted the *DHX15* promoter activity. ChIP assay revealed that p65 bound to the promoter region of *DHX15* (Fig. 6J). Further EMSA experiments performed with nuclear extracts from MCF-7 cells suggested that the complexity of p65 binding to the *DHX15* promoter existed (Fig. 6K). These results validated that p65 promotes *DHX15* transcription by binding to the promoter of *DHX15*. Considering the activation of p65 could be induced by DHX15, a DHX15-p65 positive feedback loop existed in BC. Therefore, there was the possibility that circRNF10 might impair the feedback loop. We found that circRNF10 downregulation resulted in elevated DHX15 level and circRNF10 overexpression downregulated DHX15 level in BC cells (Fig. 6L, M). These results suggested that circRNF10 antagonized the DHX15-p65 feedback loop in BC.

### CircRNF10 was a suppressor of BC growth in vivo

**Fig. 7:**
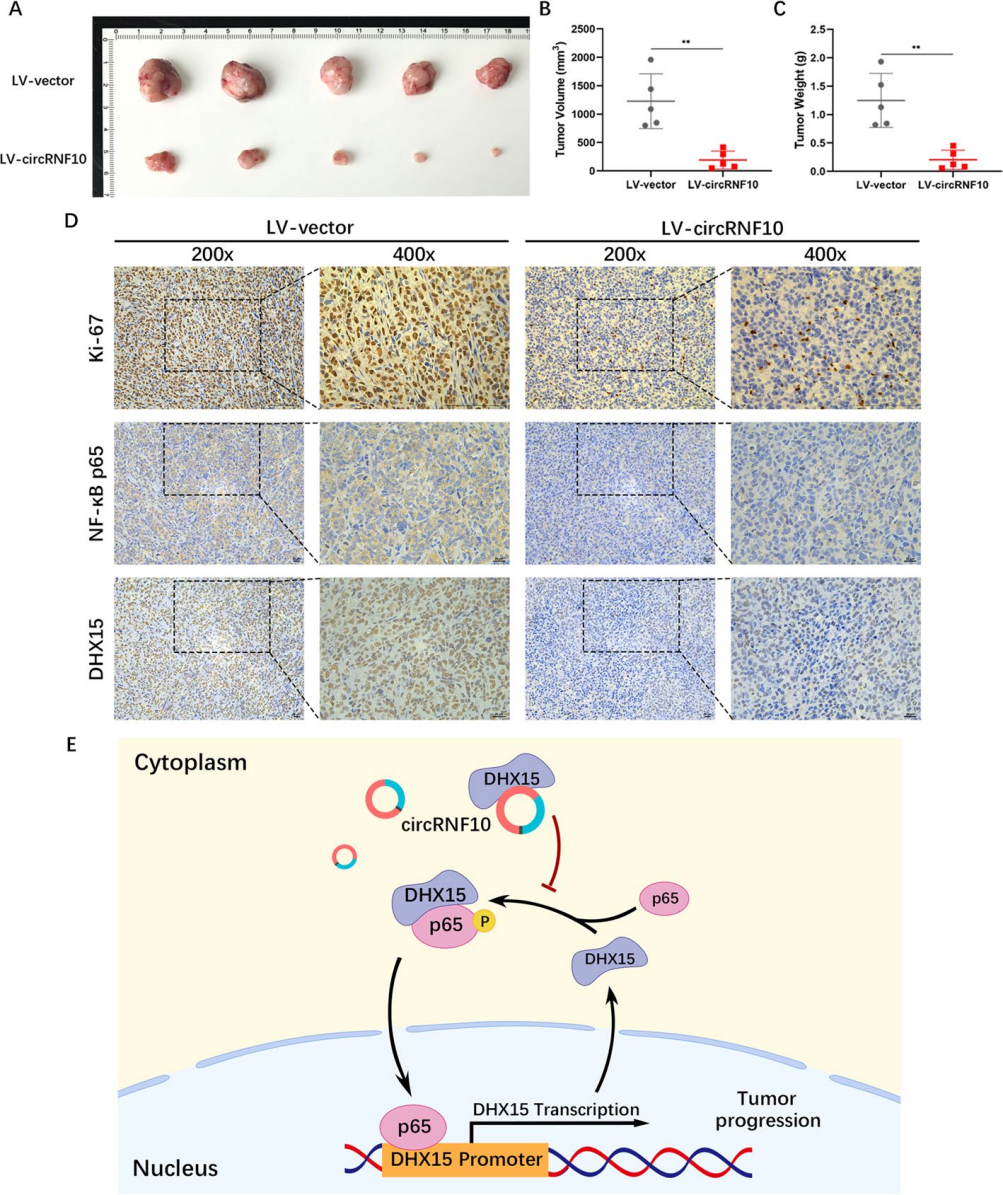
CircRNF10 suppressed BC growth in vivo. **A** Photograph of tumors in female BALB/c nude mice injected with LV-vector or LV-circRNF10 MDA-MB-231 cells (*N* = 5 for each group). **B** The calculated volume of tumors in LV-vector group and LV-circRNF10 group. **C** The weight of tumors in LV-vector group and LV-circRNF10 group. **D** Representative IHC images showing the expression of Ki-67, p65, and DHX15 in tumors of LV-vector group and LV-circRNF10 group. **E** Schematic illustration showing the mechanism of circRNF10 in inhibiting BC progression. Error bars represent the mean ± SD. *P < 0.05, **P < 0.01, ***P < 0.001, ****P < 0.0001

To validate the suppressive role of circRNF10 on BC in vivo, xenograft experiment was performed by injecting cells subcutaneously into female BALB/c nude mice after establishing MDA-MB-231 cells that stably expressed LV-vector or LV-circRNF10 (Fig. 7A). The body weight of mice in each group was recorded (Additional file 2: Fig S5). The volume and weight of tumor were significantly lower in LV-circRNF10 group than in LV-vector group, indicating that circRNF10 inhibited BC growth in vivo (Fig. 7B, C). Additionally, IHC results showed that overexpression of circRNF10 reduced Ki-67, p65, and DHX15 levels in BC tumor, which were consistent with the role circRNF10 played in vitro in BC (Fig. 7D).

Taken together, circRNF10 inhibited BC growth by antagonizing DHX15-p65 positive feedback loop through circRNF10-DHX15 interaction (Fig. 7E).

## Discussion

CircRNAs have been shown to be abnormally expressed and involved in the growth and progression of various malignancies [29, 30]. Their potential to become the biomarker and therapeutic targets of malignancies was reported recent years [4, 31, 32]. Here we reported that circRNF10 was downregulated in BC, and the lower expression was related to poorer prognosis in patients with BC, indicating that circRNF10 might have the potential to become a diagnostic and prognostic biomarker of BC. This hypothesis needs to include plenty of patients with BC and then be validated in retrospective and prospective cohorts. As for the biological function of circRNF10 in BC, we discovered that circRNF10 inhibited BC proliferation and progression. These findings were consistent with previous studies, which showed that circRNF10 inhibited tumorigenicity by sponging miR-934 and miR-942-5p in BC [15, 16]. Considering the inhibitory effect of circRNF10 on BC, it raised the possibility that circRNF10 might become an agent for BC treatment. Nowadays, the circRNA expression cassettes could be delivered to cells, or even specific organelles, via encapsulation with lipid or nanoparticles [33]. Whether circRNF10 could be delivered into BC cells via such ways still requires more exploration. Additionally, the safety, efficiency, and specificity of circRNF10 to become a therapeutic agent still needs further investigation.

The regulatory mechanisms of circRNAs were closely related to their subcellular localization [30]. As for the cytoplasmic circRNAs, they mostly acted as a sponge of miRNAs [34] and decoys of proteins [35]. Previous studies have explored the role of circRNF10 as an miRNA sponge [15, 16]. Differing from these studies, we focused on the potential of circRNF10 in binding proteins and discovered a novel role in which it could bind to DHX15 in BC by interacting with the RecA2 domain. The oncoprotein DHX15 has been shown to be an ATP-dependent RNA helicase [36] and participates in the process of alternative splicing [37, 38] and ribosome biogenesis [39–41]. In recent years, several studies indicated that DHX15 could regulate the NF-κB signaling pathway [26, 27, 42]. Therefore, we hypothesized that circRNF10-DHX15 interaction played a role in regulating the NF-κB signaling pathway. Given that the activation of p65 mainly relies on its phosphorylation and nuclear translocation [43], we determined the impact of circRNF10 and DHX15 on p-p65 (Ser536) level and the subcellular localization of p65 in BC cells and validated that circRNF10 and DHX15 were an inhibitor and an activator of p65, respectively. Interestingly, we discovered that DHX15 could interact with p65, which was reported for the first time, and might be a novel mechanism of p65 activation. According to previous studies on the structure of DHX15, the CTD is located on the RNA-binding surface of RecA domains and interacts with other proteins [22]. The unique structure of DHX15 raised a possibility that there might be a competitive relationship between circRNF10-DHX15 interaction and DHX15-p65 interaction. We found that DHX15-p65 interaction increased when circRNF10 expression was inhibited, which demonstrated that circRNF10 competitively suppressed the DHX15-p65 interaction in BC by interacting with DHX15.

The constitutive activation of NF-κB signaling pathway is one of the main reasons that causes tumorigenesis and tumor progression in BC [44], and p65 is a crucial transcription factor that promotes the transcription of numerous oncogenes (45–47). However, the underlying mechanism of the constitutive activation is still elusive. Surprisingly, we discovered that p65 promoted *DHX15* transcription by binding to the promoter region of *DHX15*. Thus, a DHX15-p65 positive feedback loop exists in BC, which might be a novel mechanism in the constitutive activation of NF-κB signaling pathway. Furthermore, circRNF10 antagonized the positive feedback loop by interacting with DHX15 and led to the inhibition of BC progression. It might provide a new strategy in impairing the constitutive activation of NF-κB signaling pathway.

## Conclusions

In summary, our findings demonstrated that a downregulated circRNA, namely circRNF10, antagonized the DHX15-p65 positive feedback loop by interacting with DHX15, thereby impairing the progression of BC. It provides a novel insight into the blockage of constitutive activation of NF-κB signaling pathway and raises a potential therapeutic approach for BC treatment.

## Supplementary Information

Additional file 1. The sequences of siRNAs, primers, and probes

Additional file 2. Additional figures

Additional file 3. Original data of western blots

## Data Availability

All data are available upon reasonable requests.

## Abbreviations

BC: Breast cancer
circRNA: Circular RNA
ChIP: Chromatin immunoprecipitation
co-IP: Coimmunoprecipitation
CTD: C-terminal domain
DAPI: 4′,6-Diamidino-2-Phenylindole
Del: Deletion
DHX15: DEAH (Asp-Glu-Ala-His) box helicase 15
EMSA: Electrophoretic mobility shift assay
FISH: Fluorescent in situ hybridization
GEO: Gene Expression Omnibus
IF: Immunofluorescence
IHC: Immunohistochemistry
LPS: Lipopolysaccharide
NF-κB: Nuclear factor kappa B
OD: Optical density
PCR: Polymerase chain reaction
PFS: Progression-free survival
RBP: RNA binding protein
RIP: RNA binding protein immunoprecipitation
RNase R: Ribonuclease R
RNF10: Ring finger protein 10
RT: Reverse transcription
RT-qPCR: Real-time quantitative PCR
SD: Standard deviation
siRNA: Small interfering RNA
WT: Wild-type
