# Supplementary material for: CircRNF10-DHX15 interaction suppressed breast cancer progression by antagonizing DHX15-NF-κB p65 positive feedback loop
Source: Cell Mol Biol Lett. 2023 Apr 26;28:34. doi: 10.1186/s11658-023-00448-7 (PMC10131429; doi:10.1186/s11658-023-00448-7)
Supplement: Supplementary file 1 — Additional file 1. The sequences of siRNAs, primers, and probes [file 11658_2023_448_MOESM1_ESM.pdf]

Table S1. The sequences of siRNAs.

siRNA	Sense (5'-3')	Anti-sense (5'-3')
si-NC	UUCUCCGAACGUGUCACGUTT	ACGUGACACGUUCGGAGAATT
si-circRNF10	UAGGAGCGCAUUUGUAGCCAUTT	AUGGCUACAAAUGCGCUCCUATT
circRNF10 si-2	CCAUUCAUCUAGGAGCGCAUUTT	AAUGCGCUCCUAGAUGAAUGGTT
circRNF10 si-3	UCAUCUAGGAGCGCAUUUGUATT	UACAAAUGCGCUCCUAGAUGATT
si-*DHX15*	GGACAUGUACAGAUAUCAATT	UUGAUAUCUGUACAUGUCCTT
si-*RELA*	GGCGAGAGGAGCACAGAUACC	UAUCUGUGCUCCUCUCGCCUG

Table S2. The sequences of primers.

Primers	Forward (5'-3')	Reverse (5'-3')
circRNF10	CTGATGAAGAGGGAGAAAGGGG	TCCACAACGGGTTATCTTGGC
RNF10	ATTTTAGCAACCAGTCCCGTCG	CCTCATCCCGTCTTCCACCAT
DHX15	TCACTCAACCCACTCAGCAC	TGGGGTAAGTTGGTGAACGG
RELA	ATGTGGAGATCATTGAGCAGC	CCTGGTCCTGTGTAGCCATT
DHX15 promoter	GGAGACCCAAGAAAGGTCGG	AGCTAAAATGGCGGCGG
ACTB	CATGTACGTTGCTATCCAGGC	CTCCTTAATGTCACGCACGAT
18S	TAGAGGGACAAGTGGCGTTC	CGCTGAGCCAGTCAGTGT
GAPDH	ACCTCAACTACATGGCTGAGAA	CCAGTGAGCTTCCCGTTCAG
U6	CAAATTCGTGAAGCGTTCCATAT	GCTTCACGAATTTGCGTGTCATCCTTGC

Table S3. The sequences of EMSA probes.

Probe	Sequences（5'-3'）
biotin-RELA oligo Forward	biotin-GGGACAGGAGTTCCCGCCTT
biotin-RELA oligo Reverse	biotin-AAGGCGGGAACTCCTGTCCC
RELA oligo Forward	GGGACAGGAGTTCCCGCCTT
RELA oligo Reverse	AAGGCGGGAACTCCTGTCCC
RELA mut Forward	GGGACAGCAGTACCCGCCTT
RELA mut Reverse	AAGGCGGGTACTGCTGTCCC
