# Supplementary material for: CircRNF10-DHX15 interaction suppressed breast cancer progression by antagonizing DHX15-NF-κB p65 positive feedback loop
Source: Cell Mol Biol Lett. 2023 Apr 26;28:34. doi: 10.1186/s11658-023-00448-7 (PMC10131429; doi:10.1186/s11658-023-00448-7)
Supplement: Supplementary file 2 — Additional file 2. Additional figures [file 11658_2023_448_MOESM2_ESM.pdf]

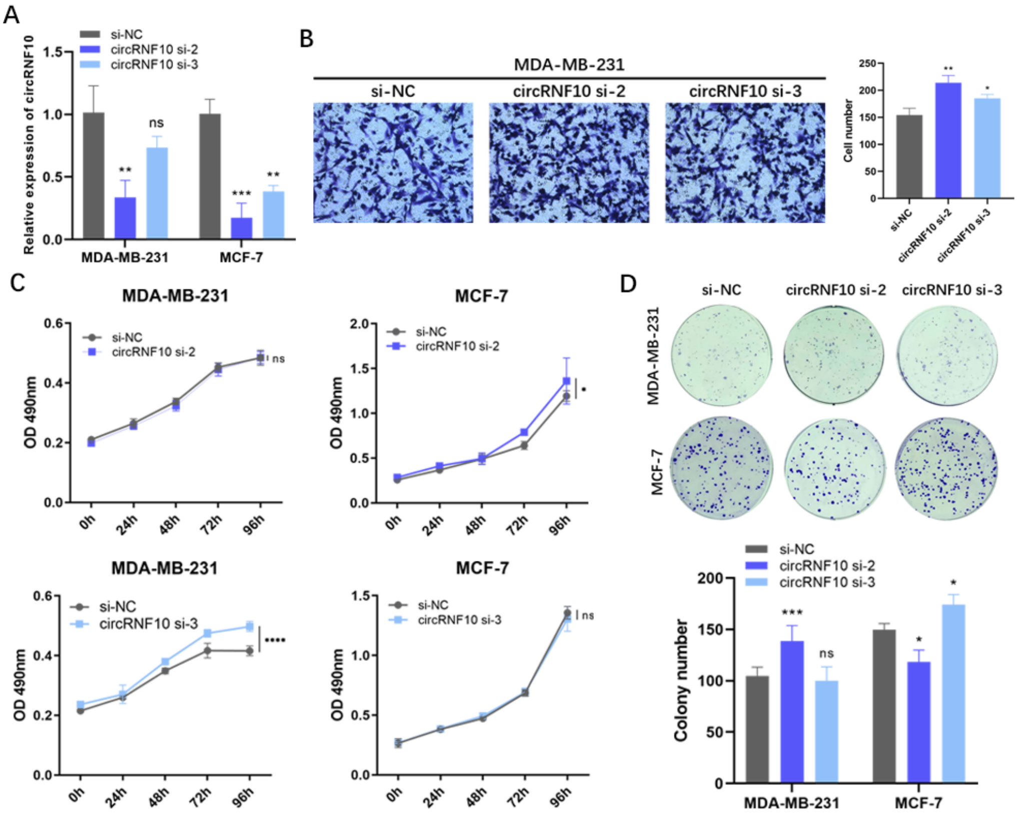

Fig. S1. The effect of circRNF10 si-2 and si-3 on BC cells. **A** RT-qPCR analysis of the efficiency of circRNF10 si-2 and si-3 in BC cells. **B** Transwell assay showing the migration of MDA-MB-231 cells transfected with circRNF10 si-2 or si-3 (left) and the number of migrated cells (right). **C** MTT assay presenting the influence of circRNF10 si-2 or si-3 on the proliferation of BC cells. **D** Colony formation assay showing the influence of circRNF10 si-2 or si-3 on the colony formation of BC cells (upper) and the number of cell colonies (lower). Error bars represent the means ± SD. **P*<0.05, ***P*<0.01, ****P*<0.001, *****P*<0.0001, ns: no significance.

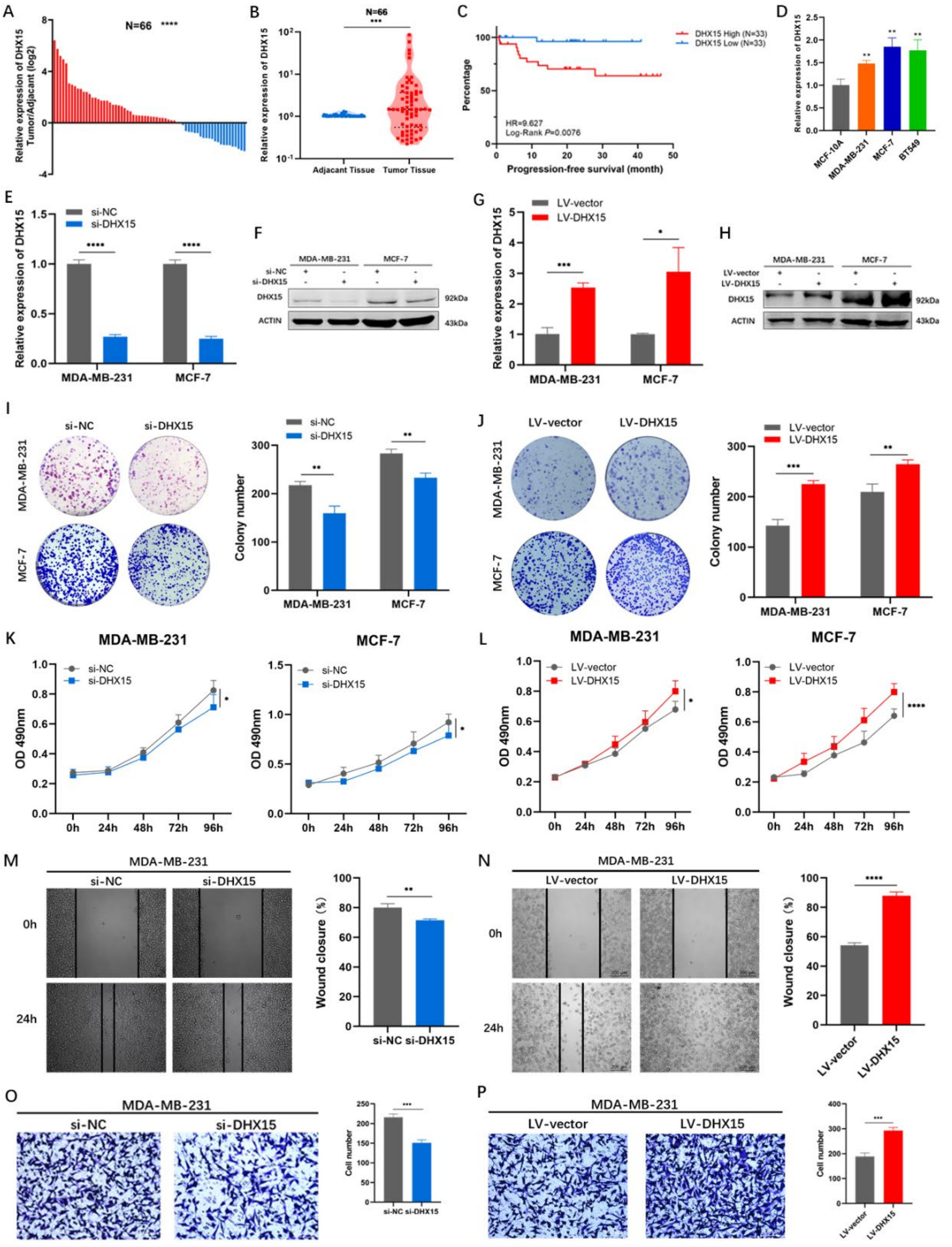

Fig. S2. DHX15 played an oncogenic role in BC. **A** RT-qPCR analysis of DHX15 expression in BC tissues presented as the ratio of tumor/adjacent (N=66). **B** RT-qPCR analysis of DHX15 expression in BC tissues and adjacent tissues (N=66). **C** K-M plot presenting PFS of high and low DHX15 expression group. **D** RT-qPCR analysis of DHX15 expression in BC cells compared to MCF-10A cells. **E** RT-qPCR analysis of si-DHX15 efficiency in BC cells. **F** Western blotting analysis of DHX15 levels in si-DHX15

group and control group of BC cells. **G** RT-qPCR analysis of LV-DHX15 efficiency in BC cells. **H** Western blotting analysis of DHX15 levels in LV-DHX15 group and control group of BC cells. **I** Colony formation assay performed in si-NC and si-DHX15 groups to evaluate the effect of inhibiting DHX15 in BC cells (left) and the number of cell colonies (right). **J** Colony formation assay performed in LV-vector and LV-DHX15 groups to evaluate the effect of overexpressing DHX15 in BC cells (left) and the number of cell colonies (right). **K** MTT assay to assess the proliferation of BC cells in si-NC group and si-DHX15 group. **L** MTT assay to assess the proliferation of BC cells in LV-vector group and LV-DHX15 group. **M** Wound-healing assay showing the migration of MDA-MB-231 cells in si-NC group and si-DHX15 group (left) and the percentage of wound closure (right). **N** Wound-healing assay showing the migration of MDA-MB-231 cells in LV-vector group and LV-DHX15 group (left) and the percentage of wound closure (right). **O** Transwell assay comparing the migration of MDA-MB-231 cells in si-DHX15 group to control (left) and the number of migrated cells (right). **P** Transwell assay comparing the migration of MDA-MB-231 cells in LV-DHX15 group to control (left) and the number of migrated cells (right). Error bars represent the means ± SD. *P<0.05, **P<0.01, ***P<0.001, ****P<0.0001.

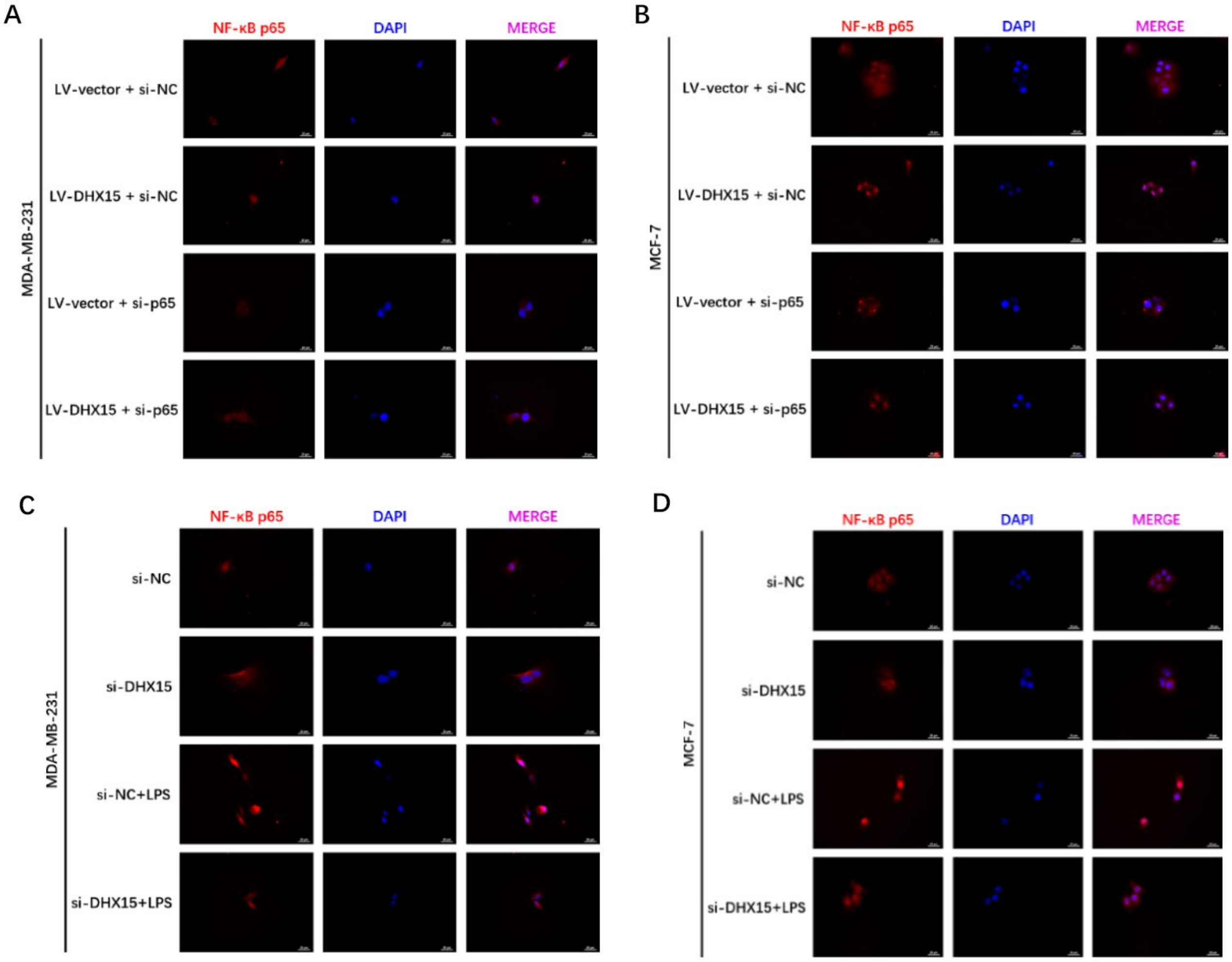

Fig. S3. The IF assays exploring the role of DHX15 in the nuclear translocation of NF-κB p65. **A** IF assay showing the effect of LV-DHX15 on the subcellular localization of p65 when p65 was suppressed in MDA-MB-231 cells. Red, p65; blue: DAPI. **B** IF assay showing the effect of LV-DHX15 on the subcellular localization of p65 when p65 was suppressed in MCF-7 cells. Red, p65; blue: DAPI. **C** IF assay showing the influence of si-DHX15 on LPS-induced p65 translocation in MDA-MB-231 cells. Red, p65; blue: DAPI. **D** IF assay showing the influence of si-DHX15 on LPS-induced p65 translocation in MCF-7 cells. Red, p65; blue: DAPI.

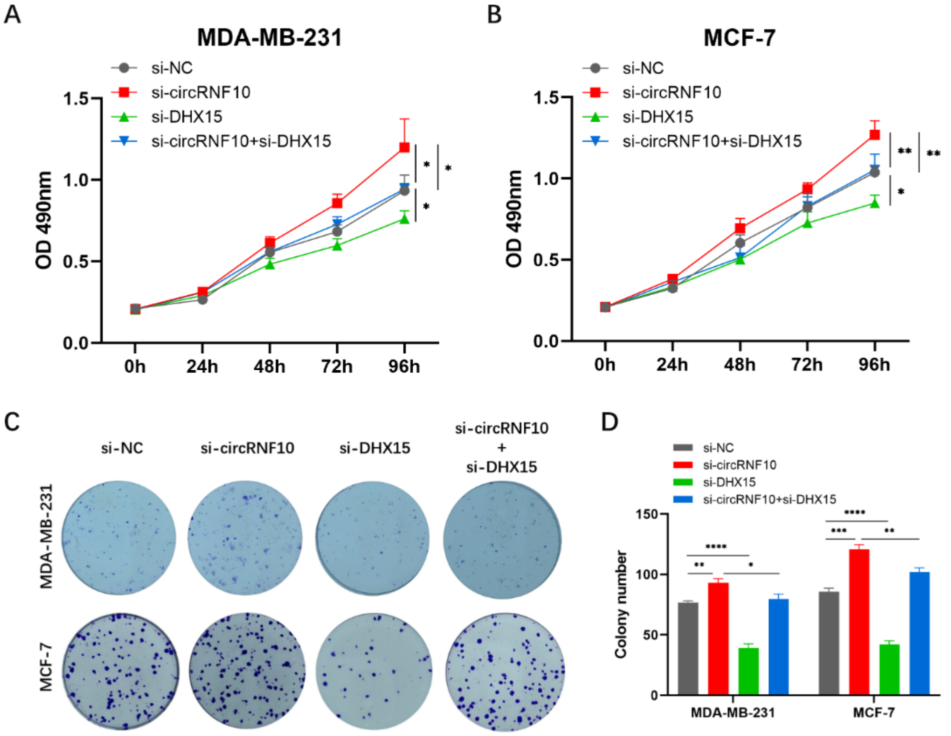

Fig. S4. The rescue experiments exploring the influence of knocking down circRNF10 on DHX15 knockdown cells. **A** MTT assay showing the influence of si-circRNF10 on the proliferation of DHX15 knockdown MDA-MB-231 cells. **B** MTT assay showing the influence of si-circRNF10 on the proliferation of DHX15 knockdown MCF-7 cells. **C** Colony formation assay showing the effect of si-circRNF10 on the proliferation of DHX15 knockdown BC cells. **D** The number of colonies. Error bars represent the means ± SD. *P<0.05, **P<0.01, ***P<0.001, ****P<0.0001.

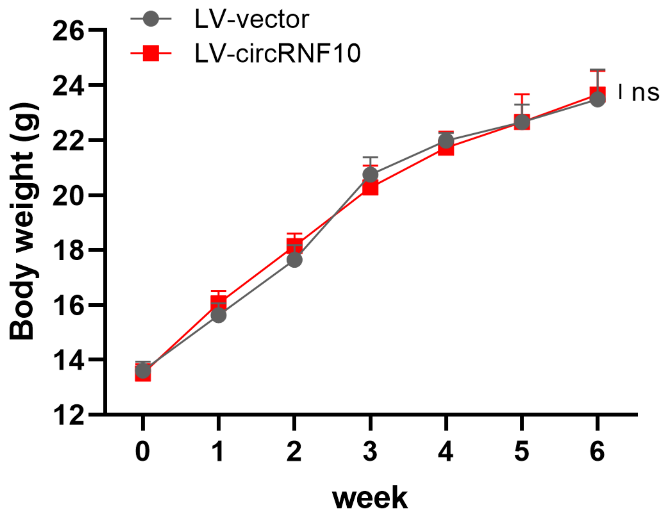

Fig. S5 The body weight of mice in xenograft experiment. Error bars represent the means ± SD. **P*<0.05, ***P*<0.01, ****P*<0.001, *****P*<0.0001, ns: no significance.
