# Supplementary material for: CircRNF10-DHX15 interaction suppressed breast cancer progression by antagonizing DHX15-NF-κB p65 positive feedback loop
Source: Cell Mol Biol Lett. 2023 Apr 26;28:34. doi: 10.1186/s11658-023-00448-7 (PMC10131429; doi:10.1186/s11658-023-00448-7)
Supplement: Supplementary file 3 — Additional file 3. Original data of western blots [file 11658_2023_448_MOESM3_ESM.pdf]

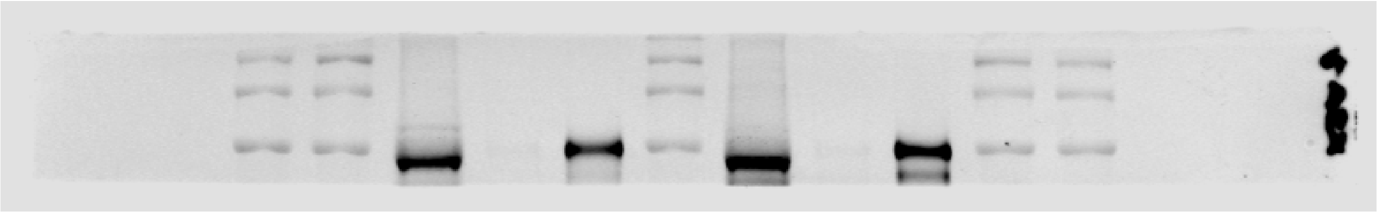

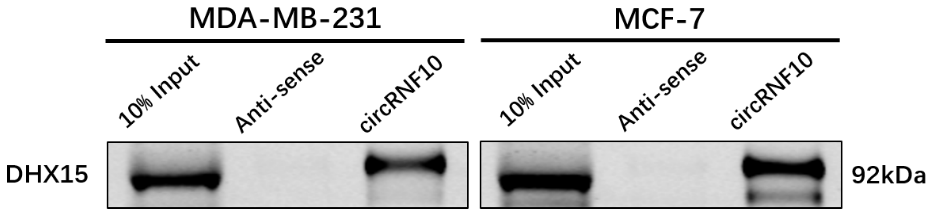
MDA-MB-231
MCF-7
10% Input
Anti-sense
circRNF10
10% Input
Anti-sense
circRNF10
DHX15
92kDa

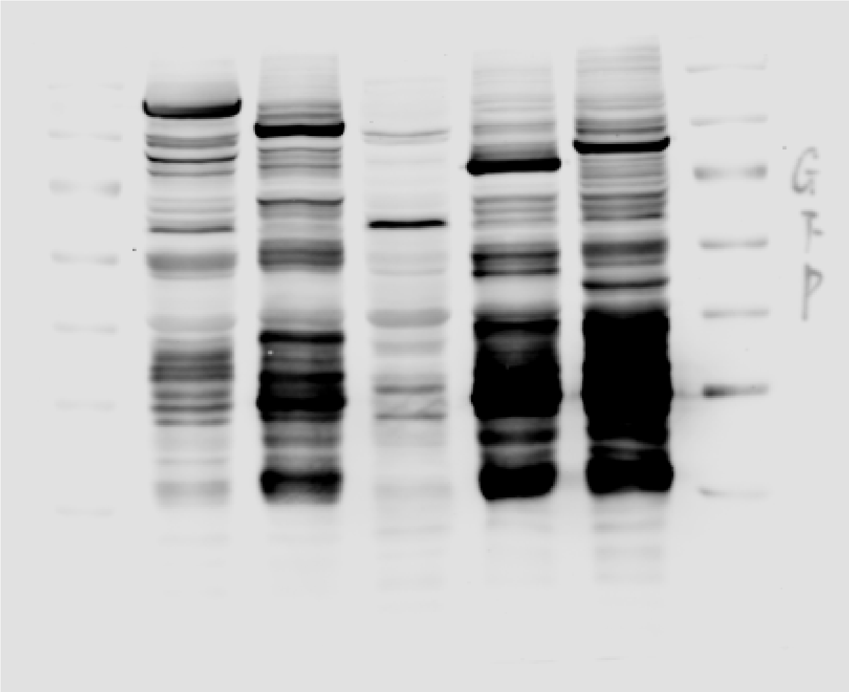

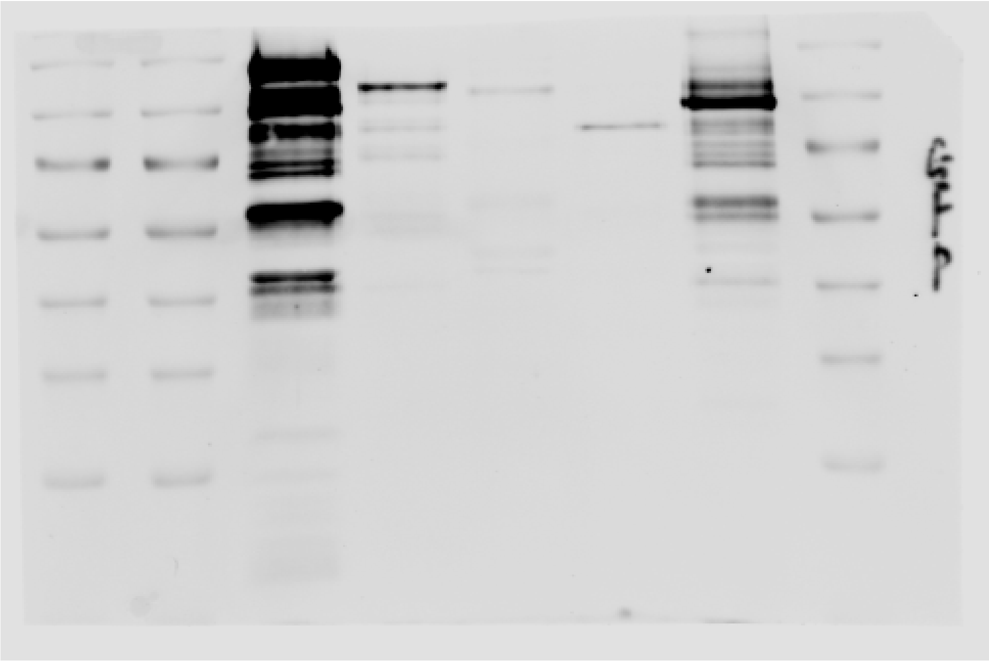

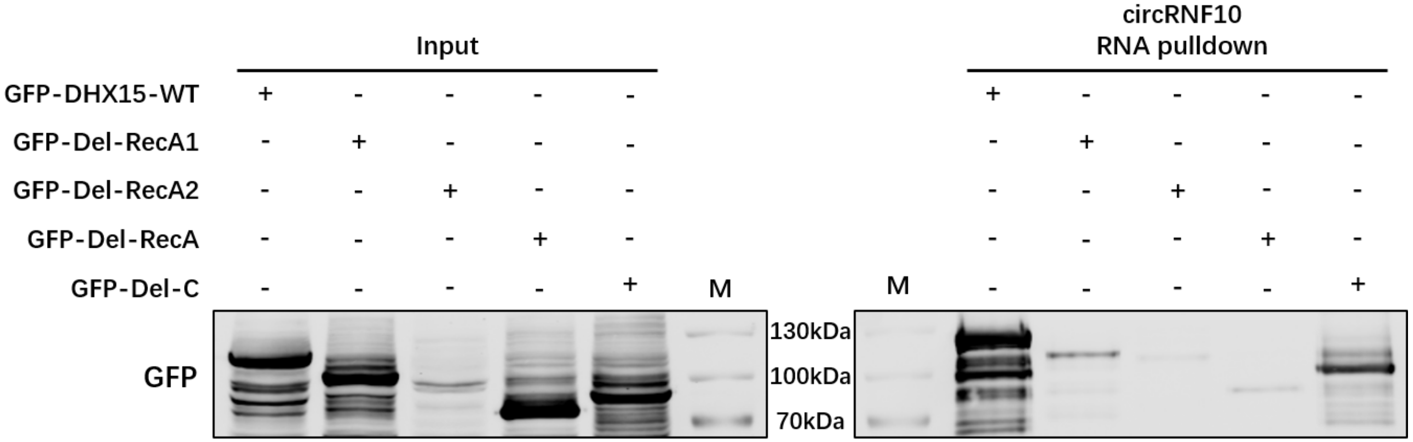
Input
circRNF10
RNA pulldown
GFP-DHX15-WT
GFP-Del-RecA1
GFP-Del-RecA2
GFP-Del-RecA
GFP-Del-C
M
M
GFP
130kDa
100kDa
70kDa

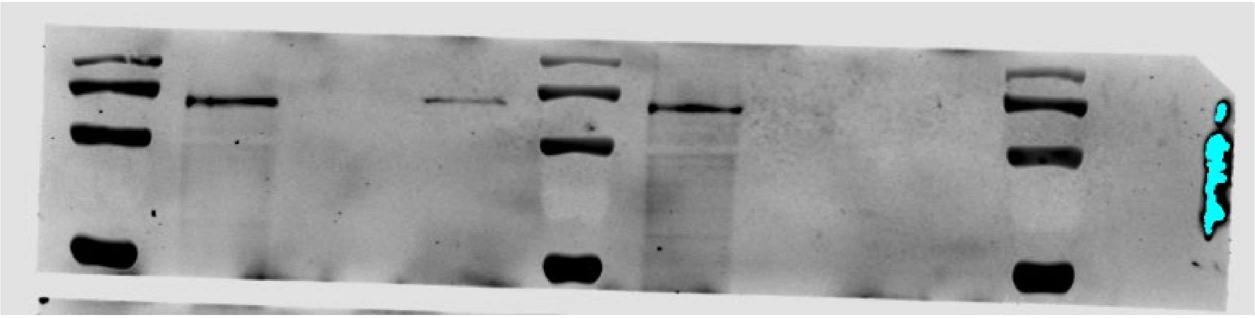

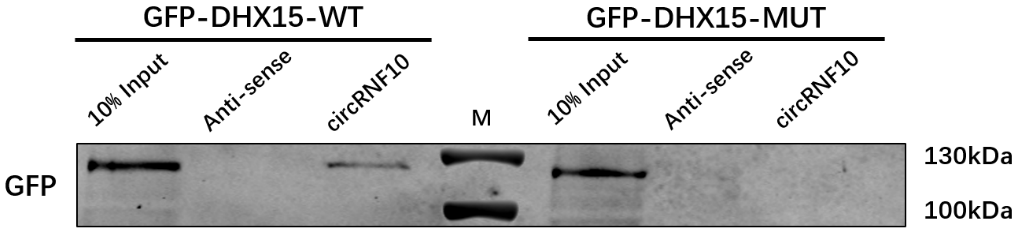
GFP-DHX15-WT
GFP-DHX15-MUT
10% Input
Anti-sense
circRNF10
M
10% Input
Anti-sense
circRNF10
GFP
130kDa
100kDa

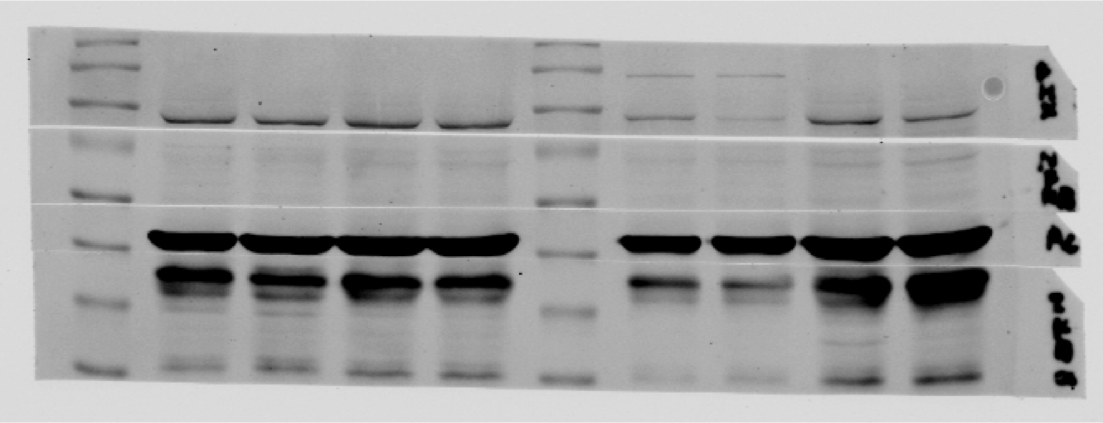

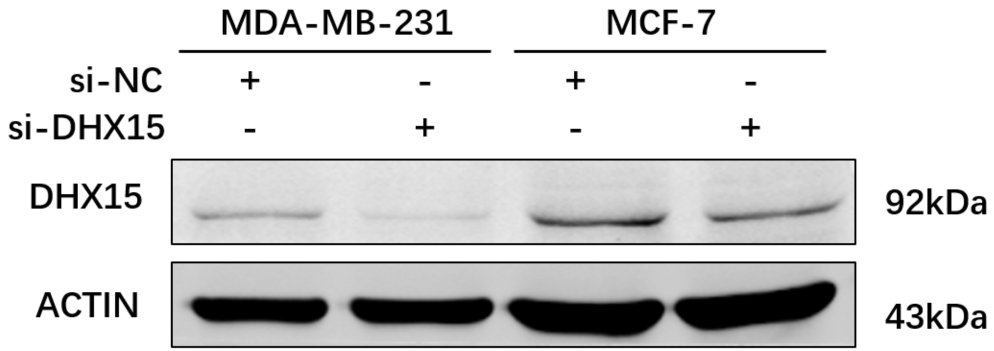
MDA-MB-231
MCF-7
si-NC + - + -
si-DHX15 - + - +
DHX15
92kDa
ACTIN
43kDa

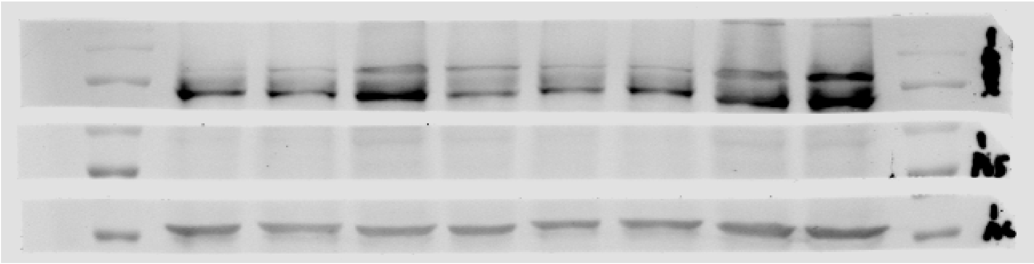

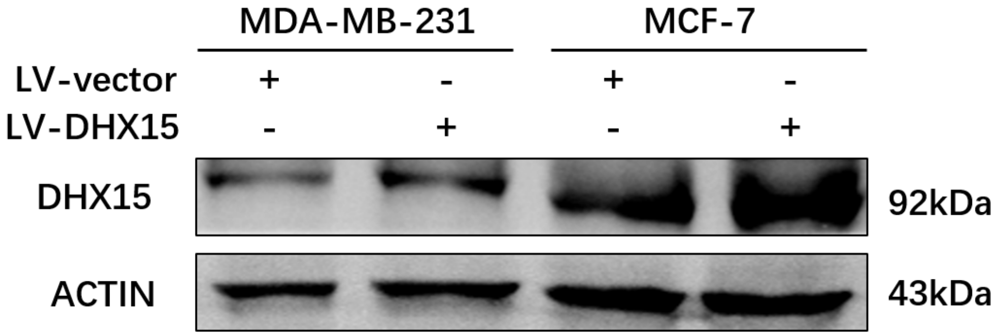
MDA-MB-231
MCF-7
LV-vector + - + -
LV-DHX15 - + - +
DHX15
92kDa
ACTIN
43kDa

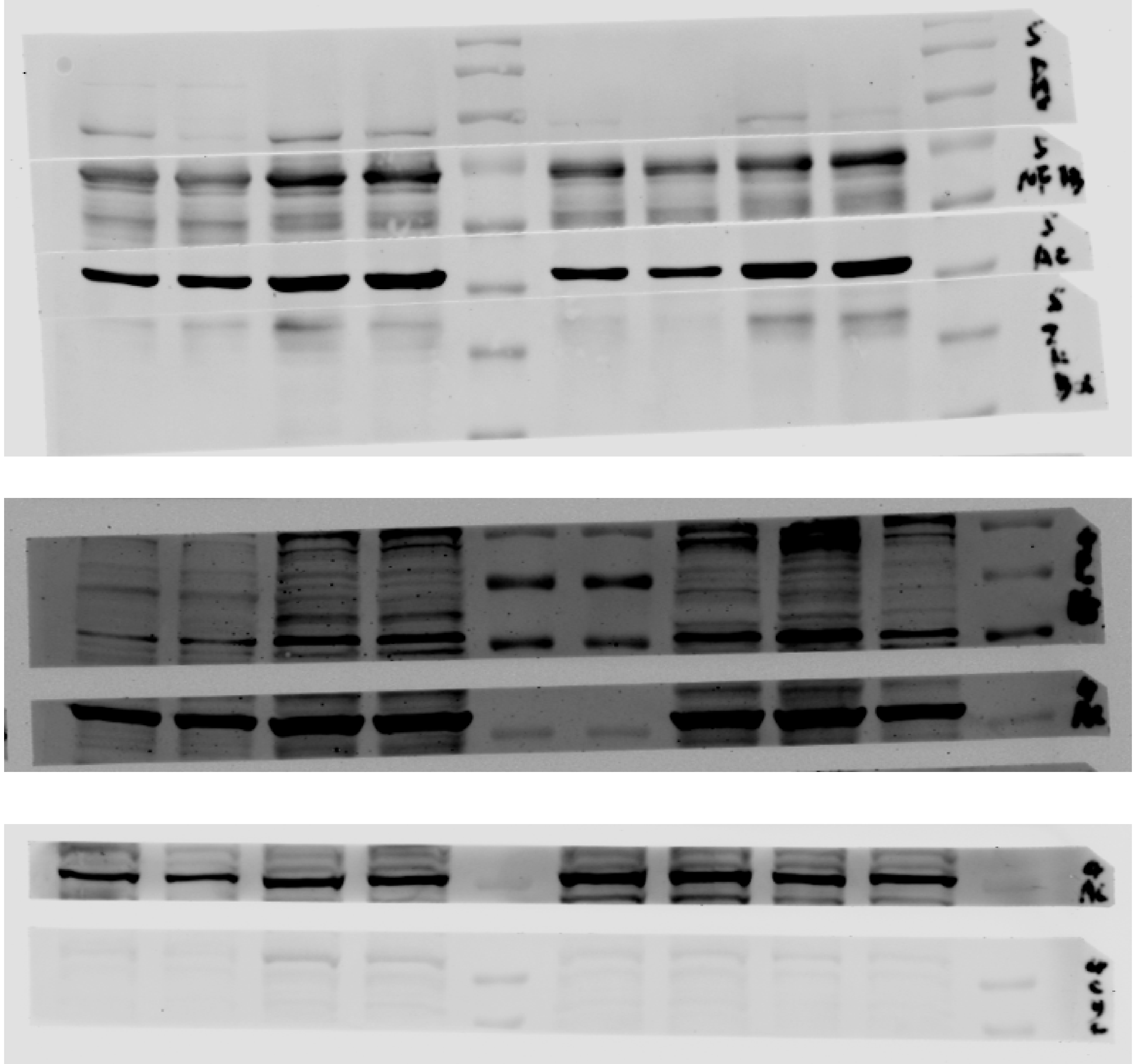

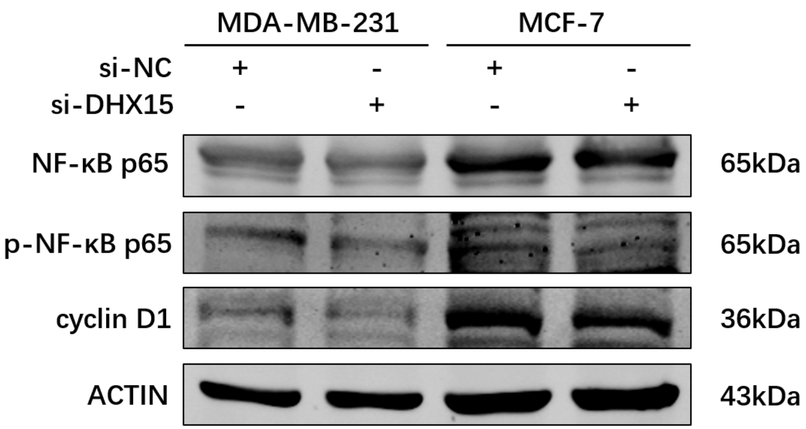
MDA-MB-231
MCF-7
si-NC + - + -
si-DHX15 - + - +
NF-κB p65
65kDa
p-NF-κB p65
65kDa
cyclin D1
36kDa
ACTIN
43kDa

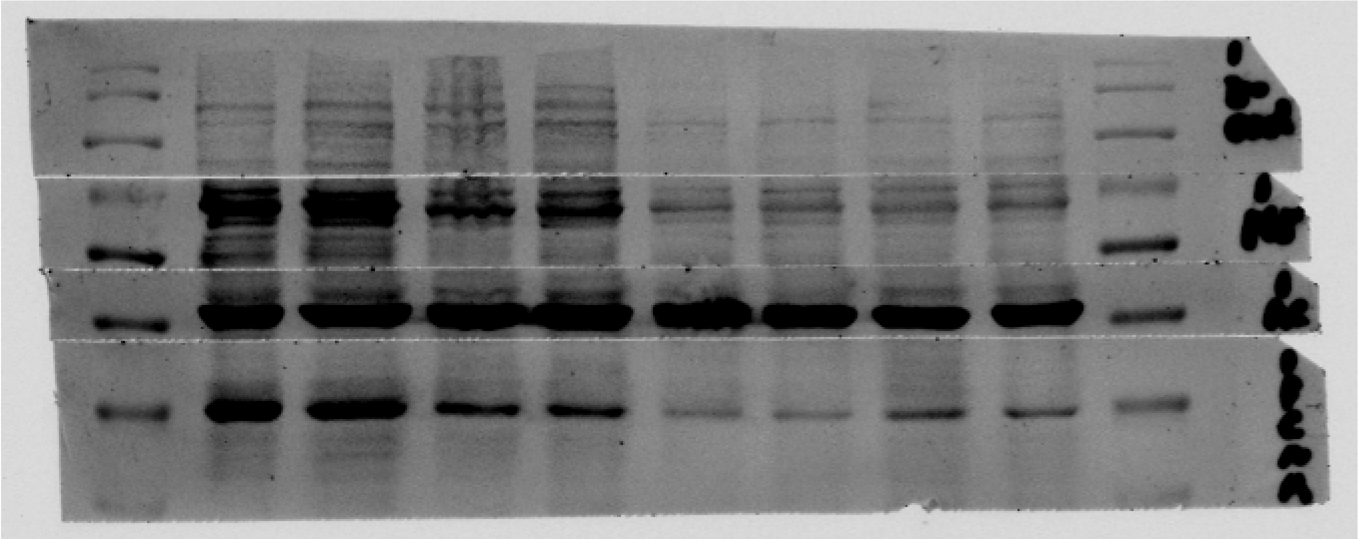

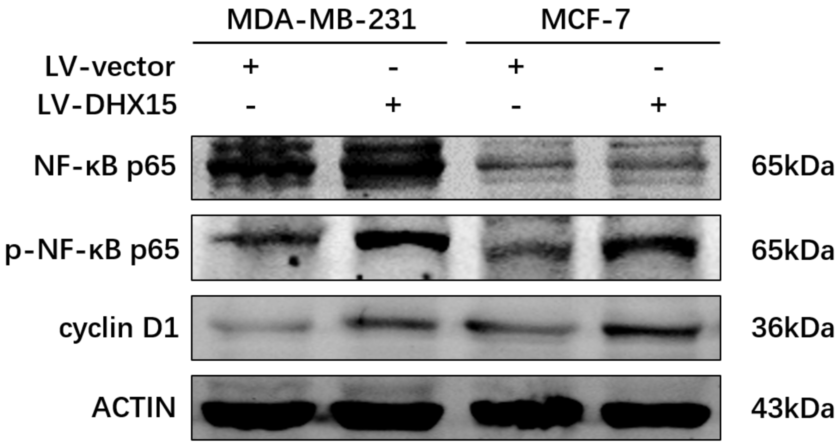
MDA-MB-231
MCF-7
LV-vector + - + -
LV-DHX15 - + - +
NF-κB p65
65kDa
p-NF-κB p65
65kDa
cyclin D1
36kDa
ACTIN
43kDa

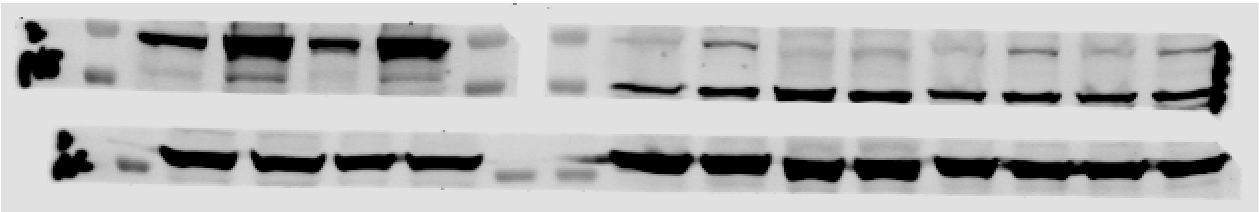

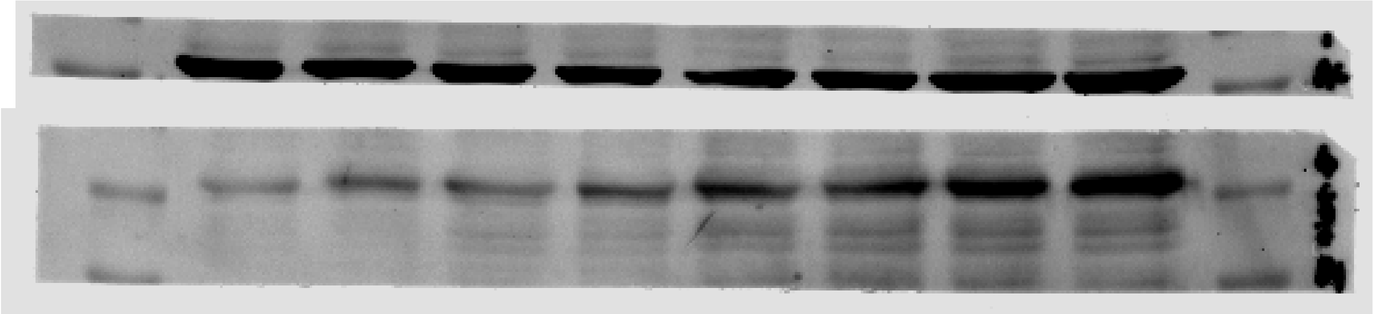

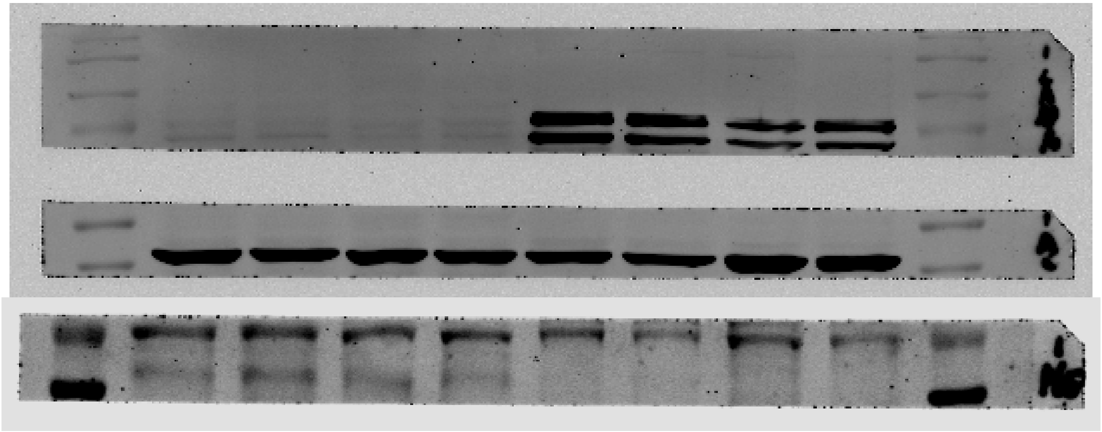

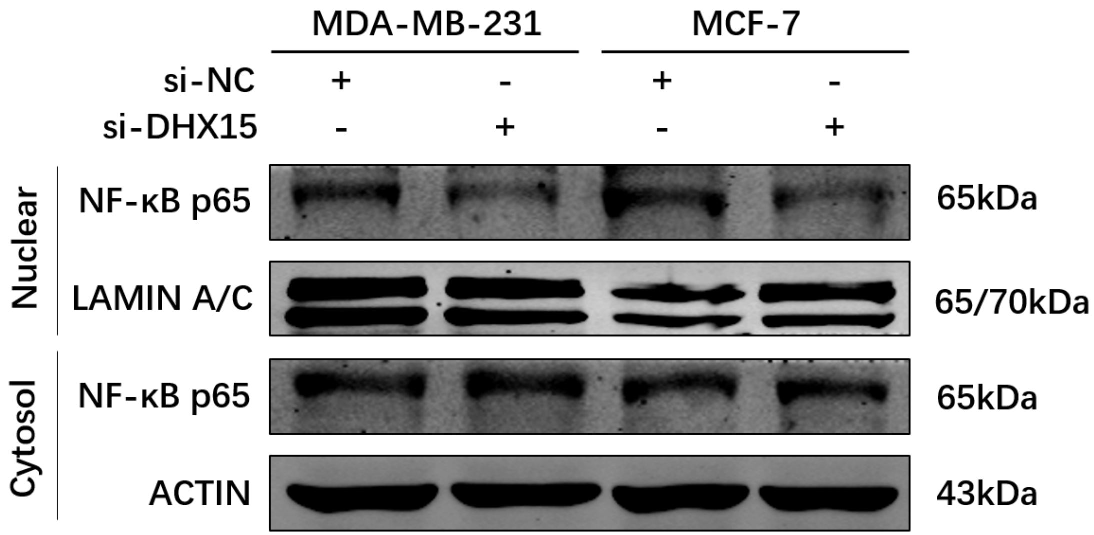
MDA-MB-231
MCF-7
si-NC + - + -
si-DHX15 - + - +
Nuclear
NF-κB p65
65kDa
LAMIN A/C
65/70kDa
Cytosol
NF-κB p65
65kDa
ACTIN
43kDa

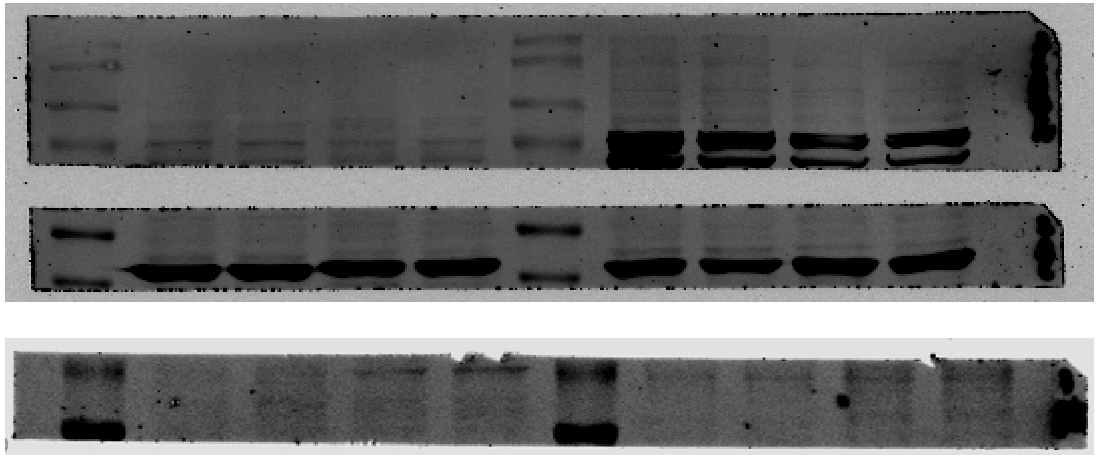

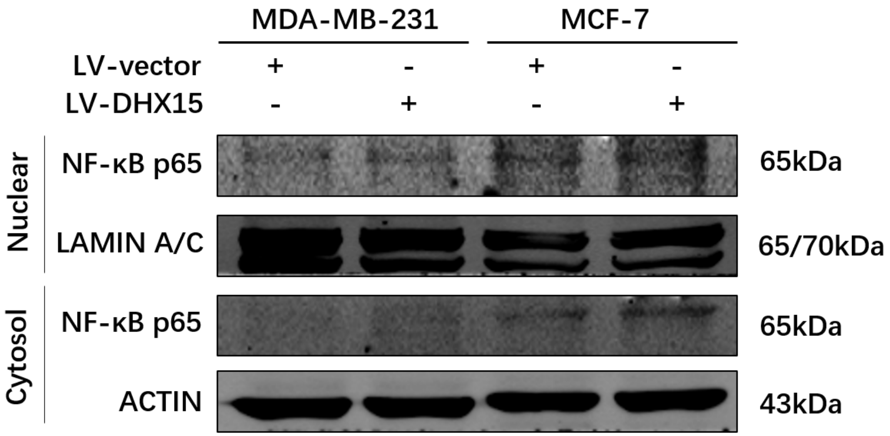
MDA-MB-231
MCF-7
LV-vector
+
-
+
-
LV-DHX15
-
+
-
+
Nuclear
NF-κB p65
65kDa
LAMIN A/C
65/70kDa
Cytosol
NF-κB p65
65kDa
ACTIN
43kDa

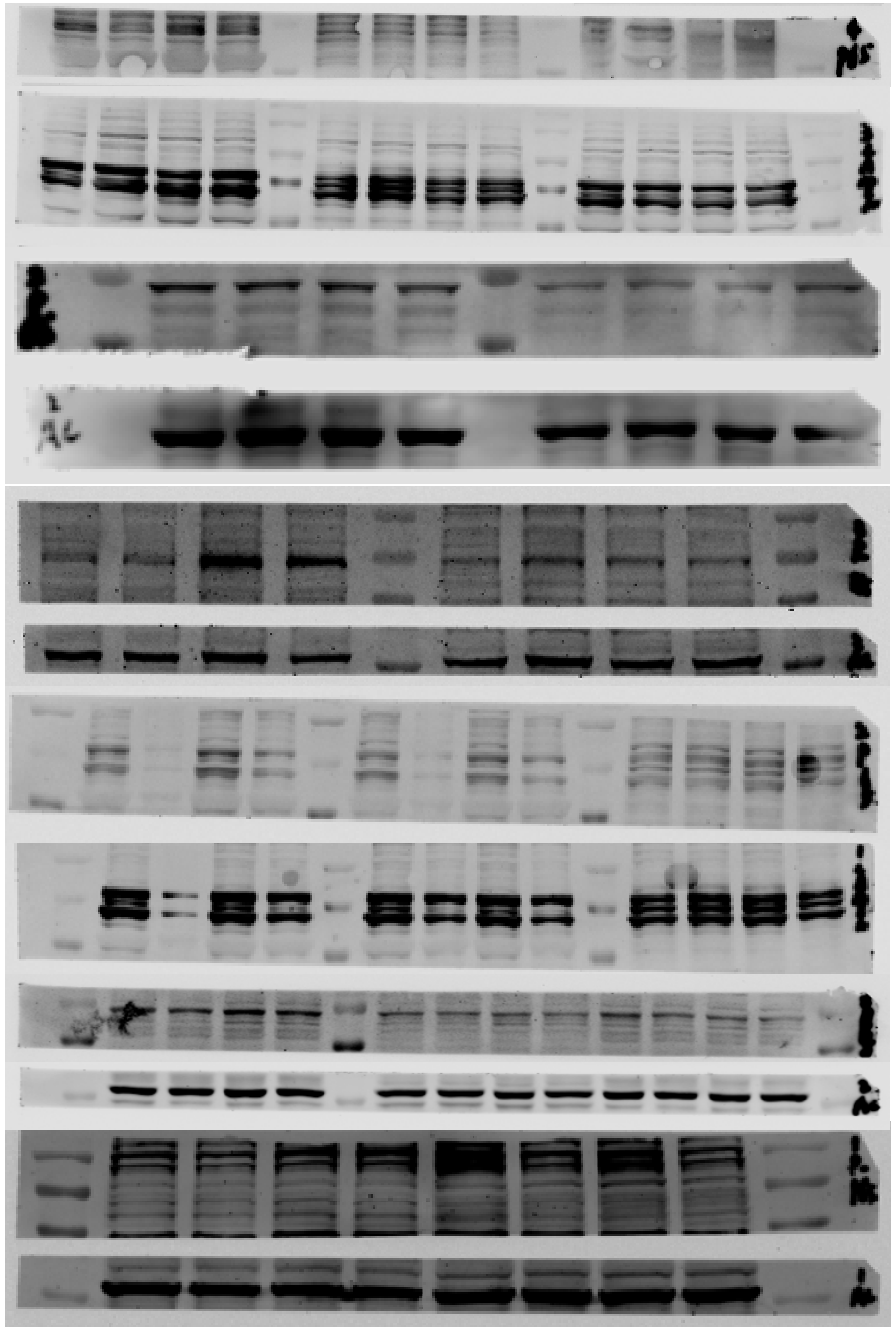

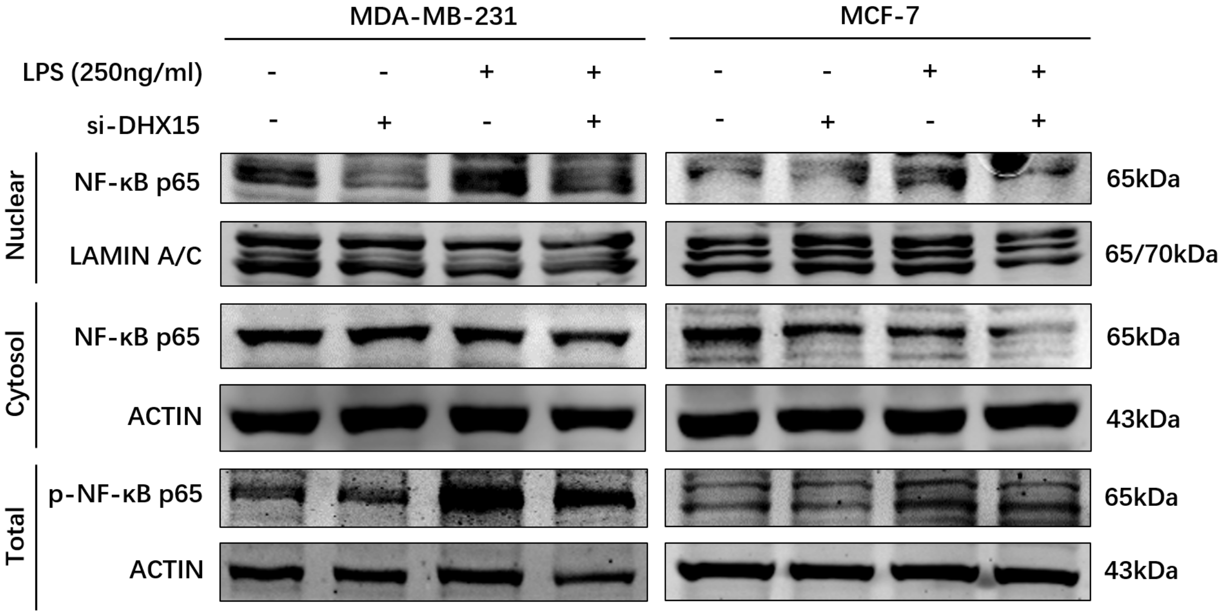
MDA-MB-231
MCF-7
LPS (250ng/ml)
si-DHX15
Nuclear
NF-κB p65
LAMIN A/C
Cytosol
NF-κB p65
ACTIN
Total
p-NF-κB p65
ACTIN
65kDa
65/70kDa
65kDa
43kDa
65kDa
43kDa

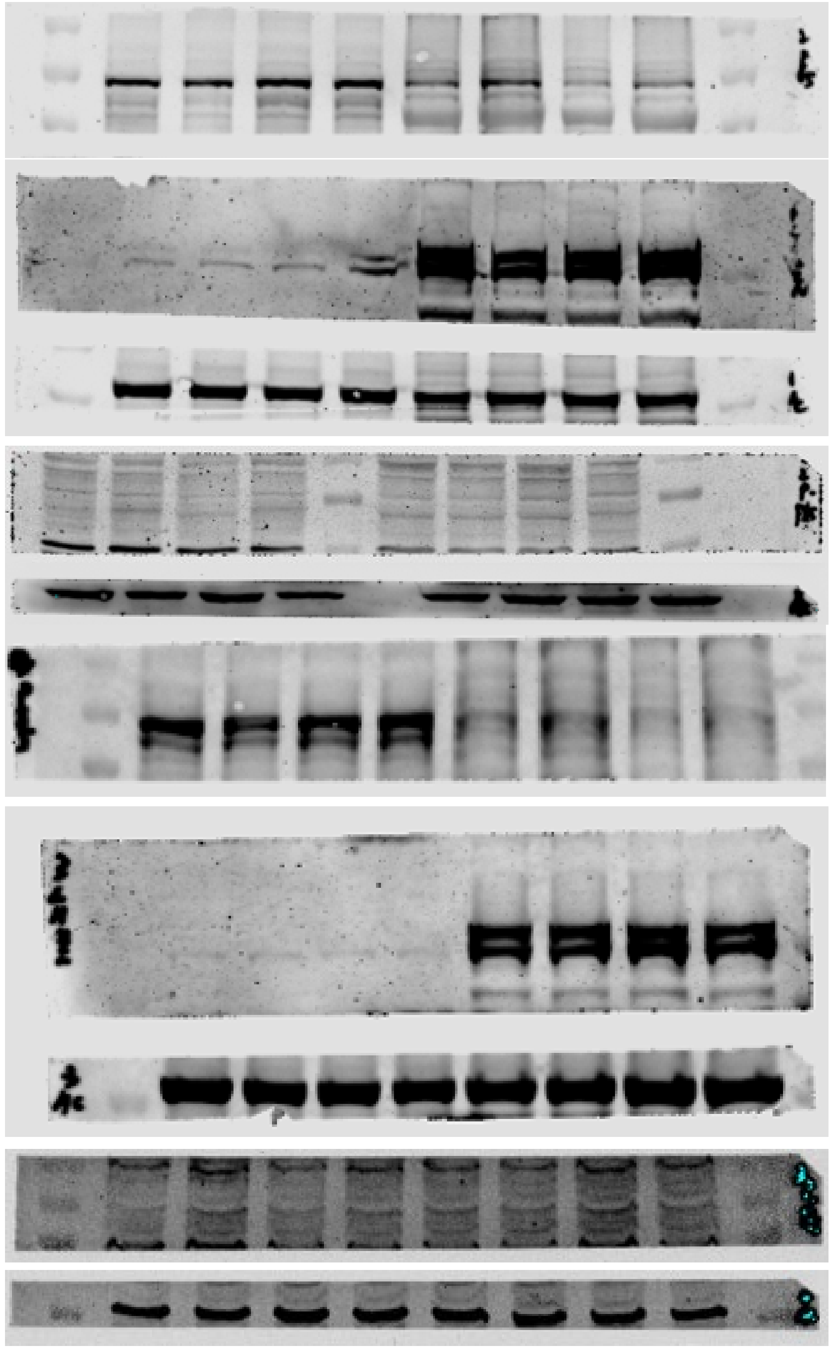

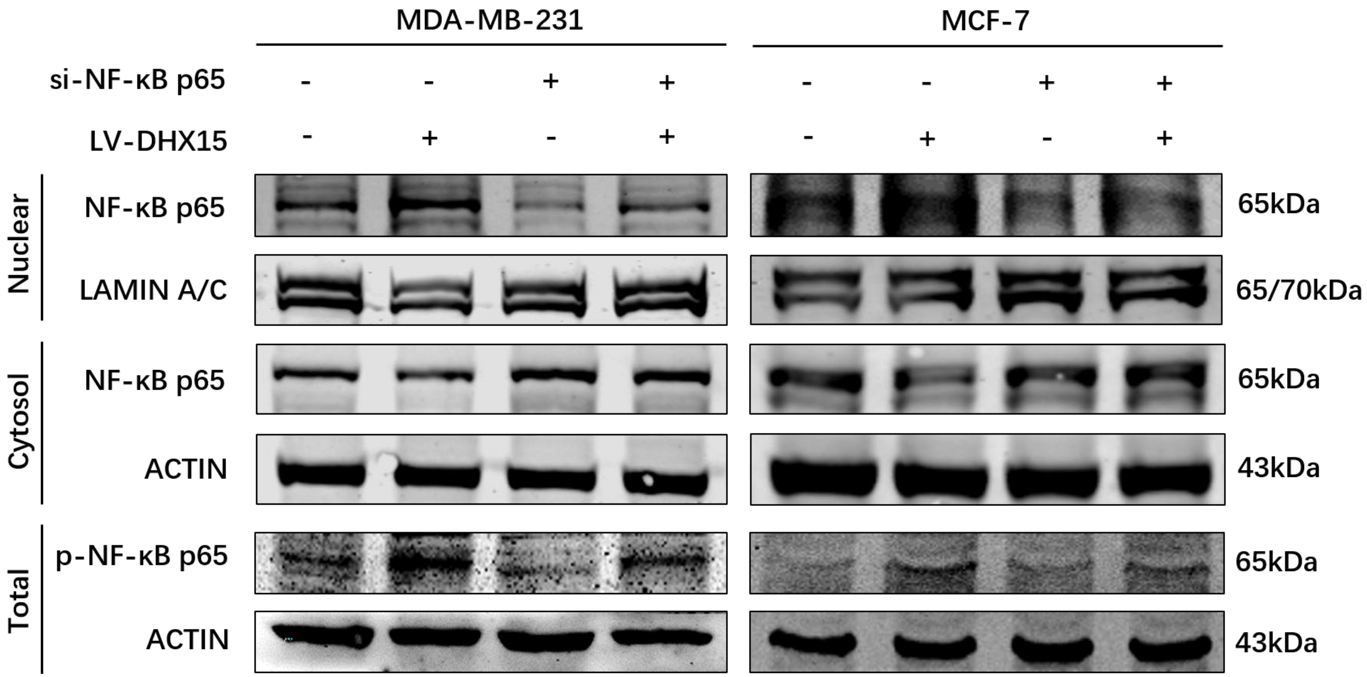
MDA-MB-231
MCF-7
si-NF-κB p65
LV-DHX15
Nuclear
NF-κB p65
LAMIN A/C
Cytosol
NF-κB p65
ACTIN
Total
p-NF-κB p65
ACTIN
65kDa
65/70kDa
65kDa
43kDa
65kDa
43kDa

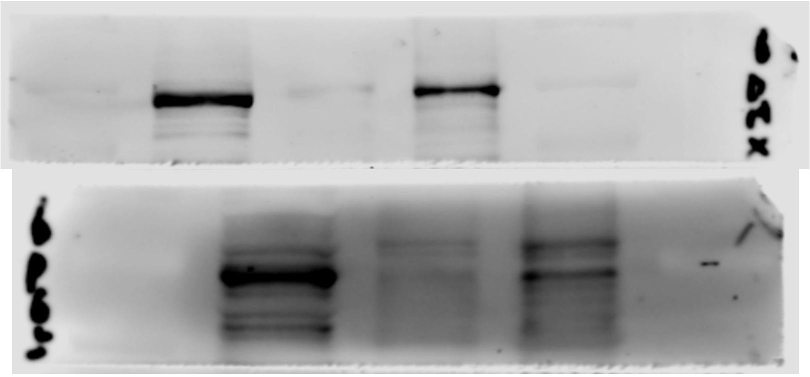

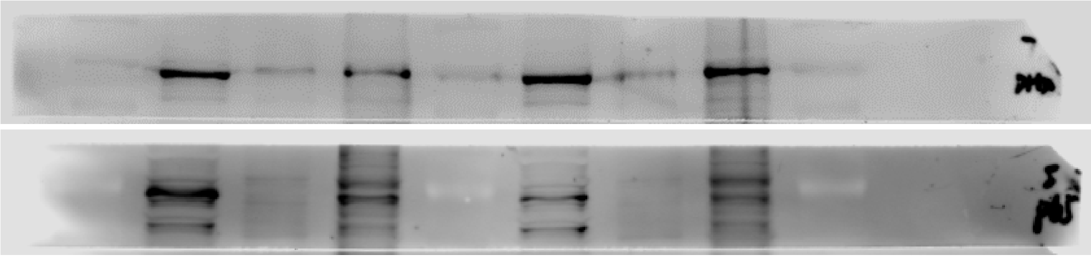

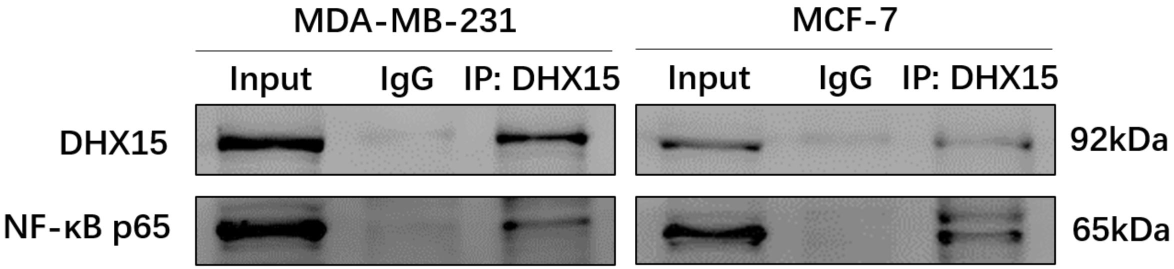
MDA-MB-231
MCF-7
Input
IgG
IP: DHX15
Input
IgG
IP: DHX15
DHX15
NF-κB p65
92kDa
65kDa

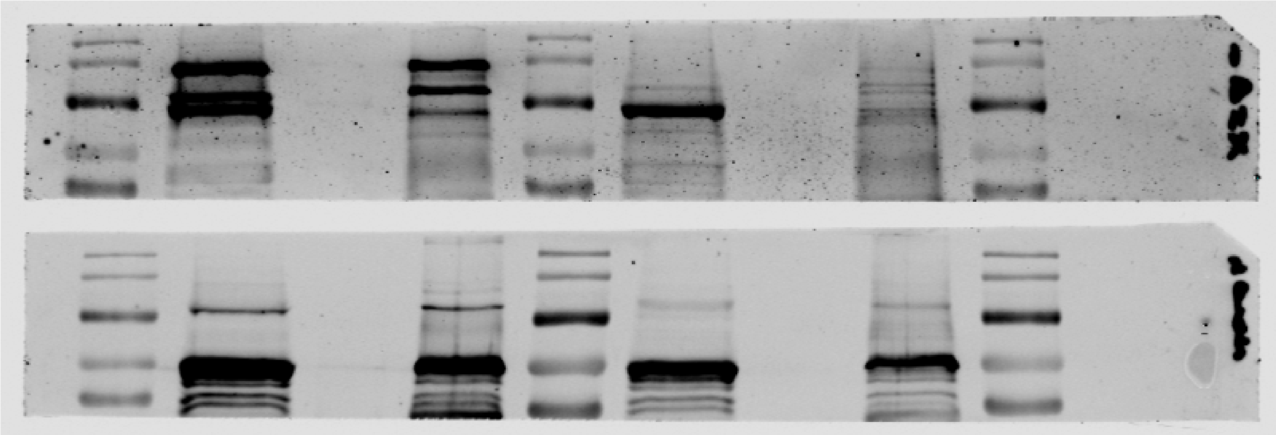

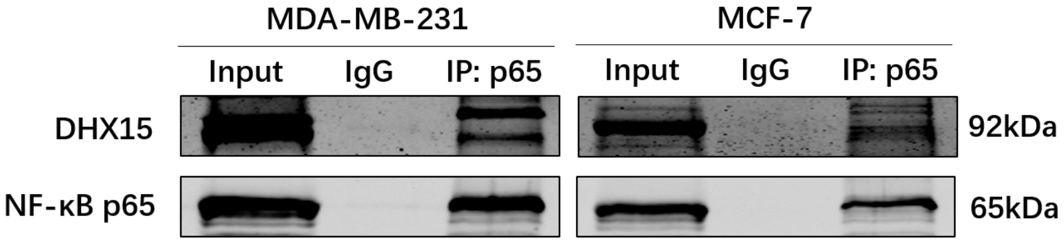
MDA-MB-231
MCF-7
Input
IgG
IP: p65
Input
IgG
IP: p65
DHX15
NF-κB p65
92kDa
65kDa

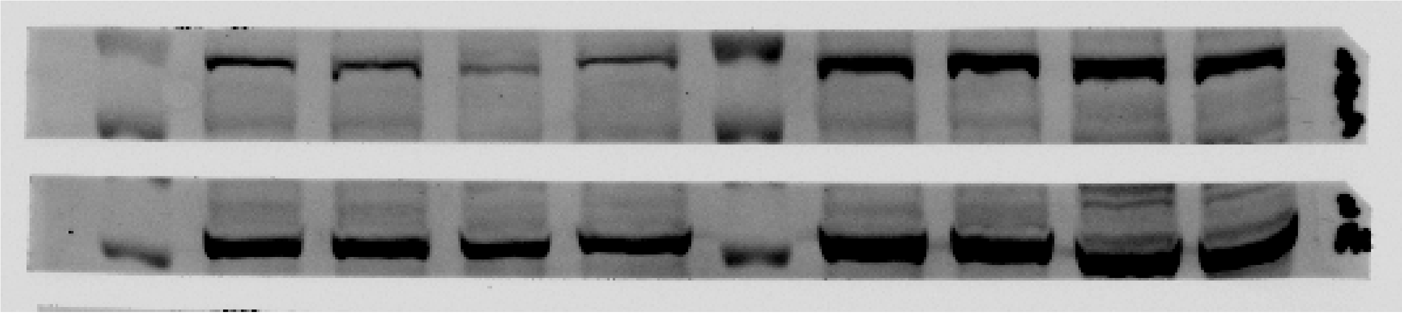

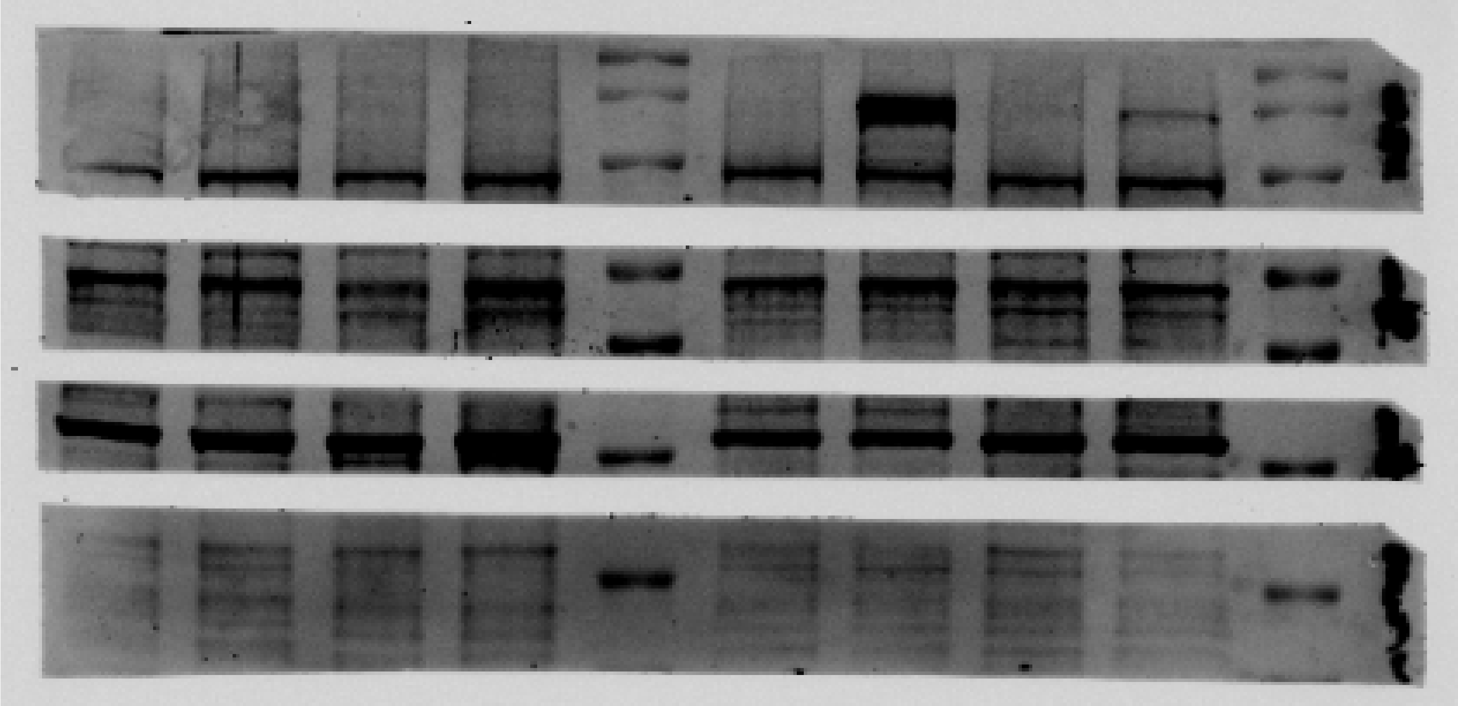

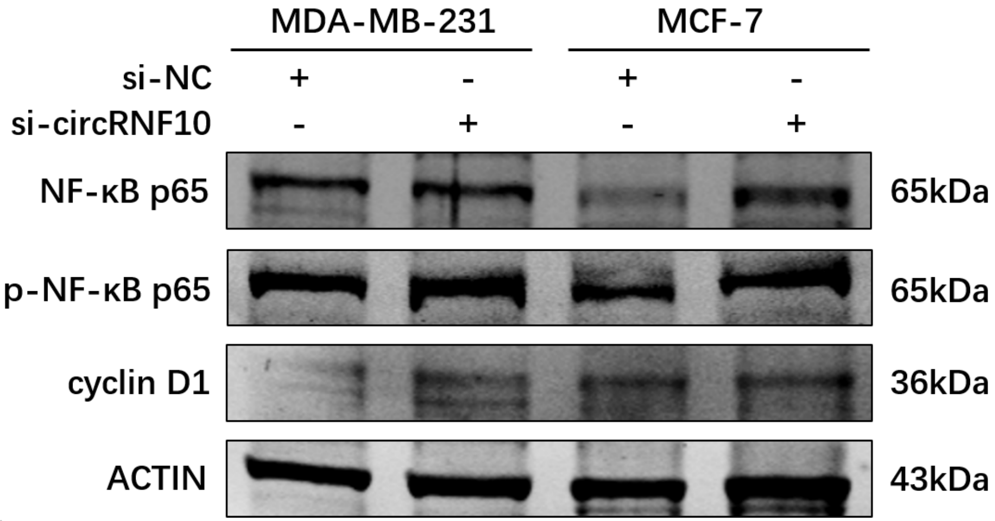
MDA-MB-231
MCF-7
si-NC
+
-
+
-
si-circRNF10
-
+
-
+
NF-κB p65
65kDa
p-NF-κB p65
65kDa
cyclin D1
36kDa
ACTIN
43kDa

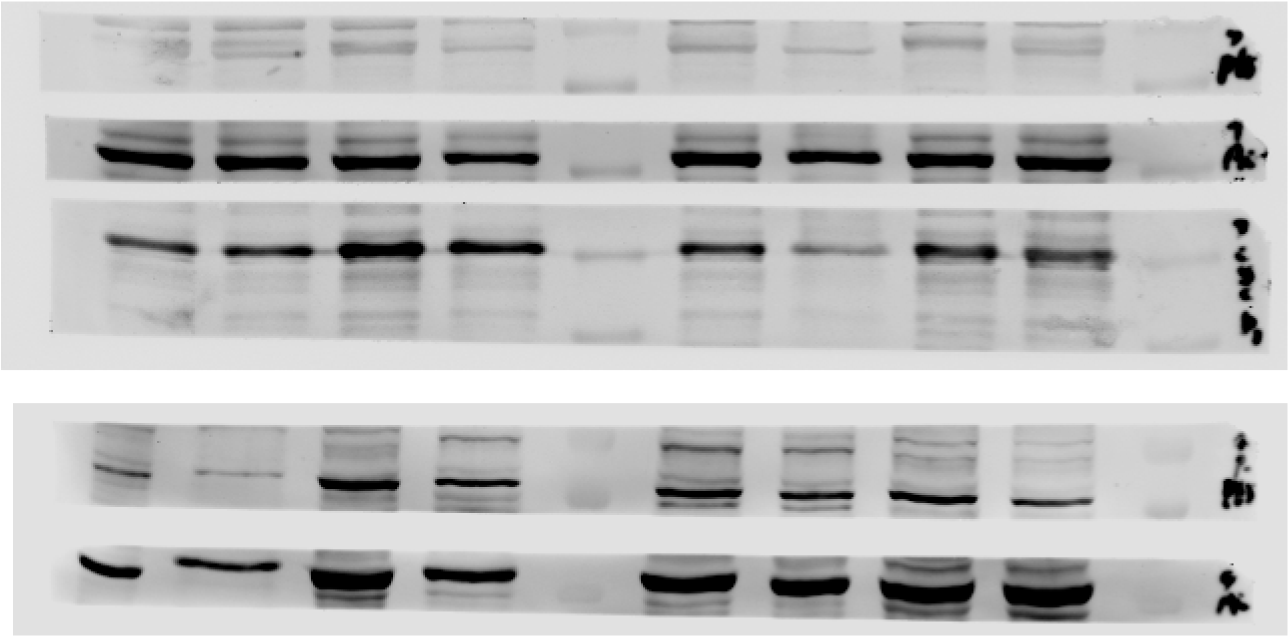

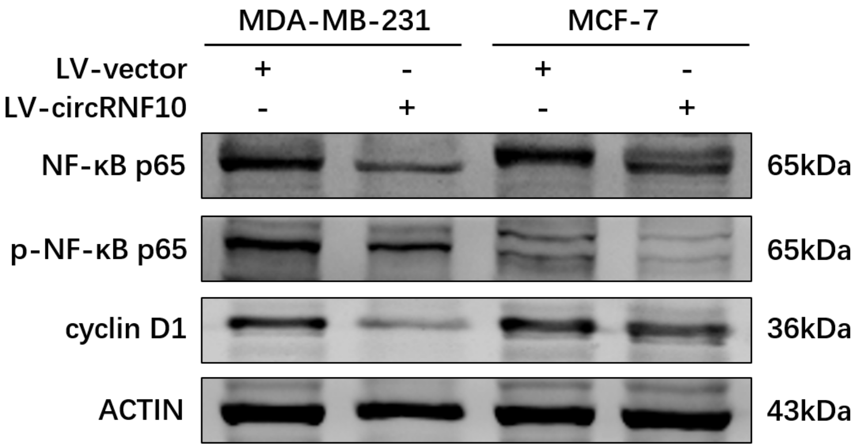
MDA-MB-231
MCF-7
LV-vector + - + -
LV-circRNF10 - + - +
NF-κB p65
65kDa
p-NF-κB p65
65kDa
cyclin D1
36kDa
ACTIN
43kDa

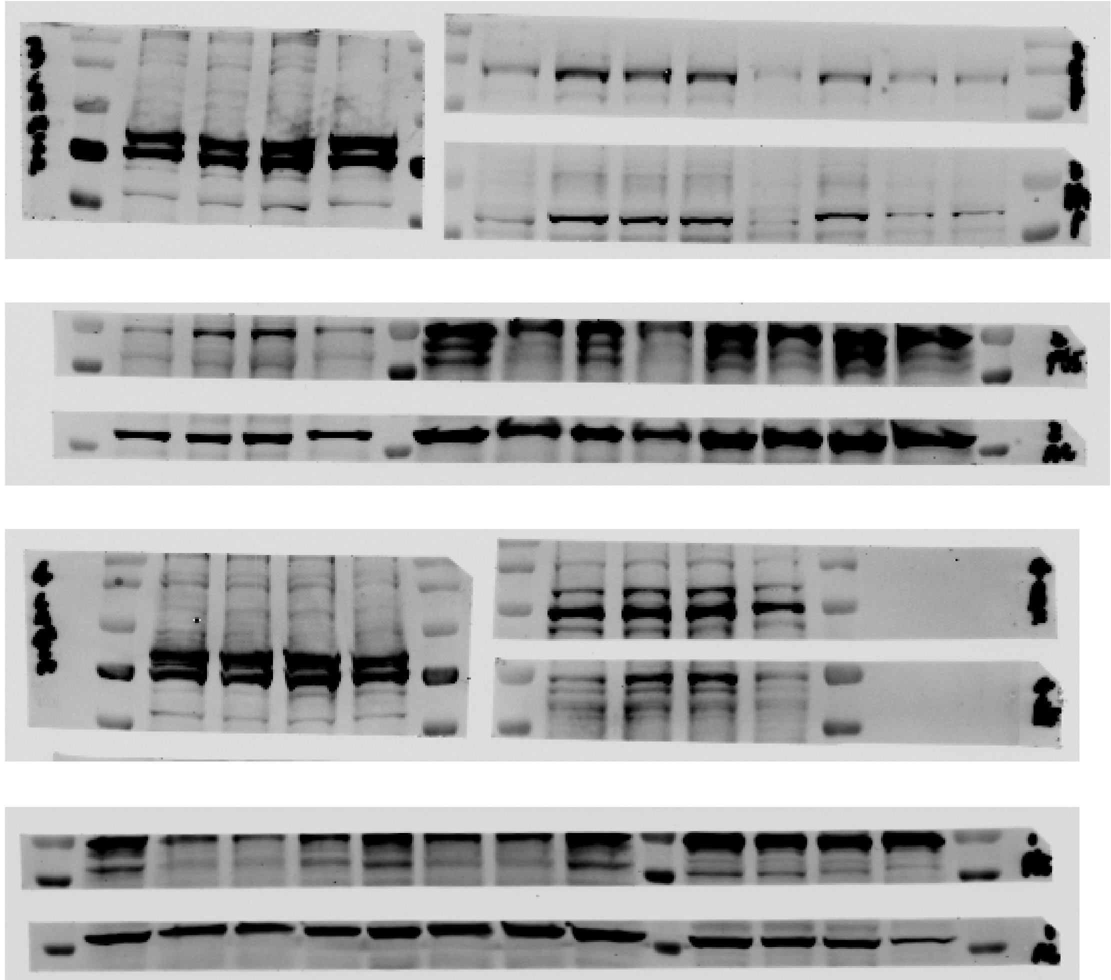

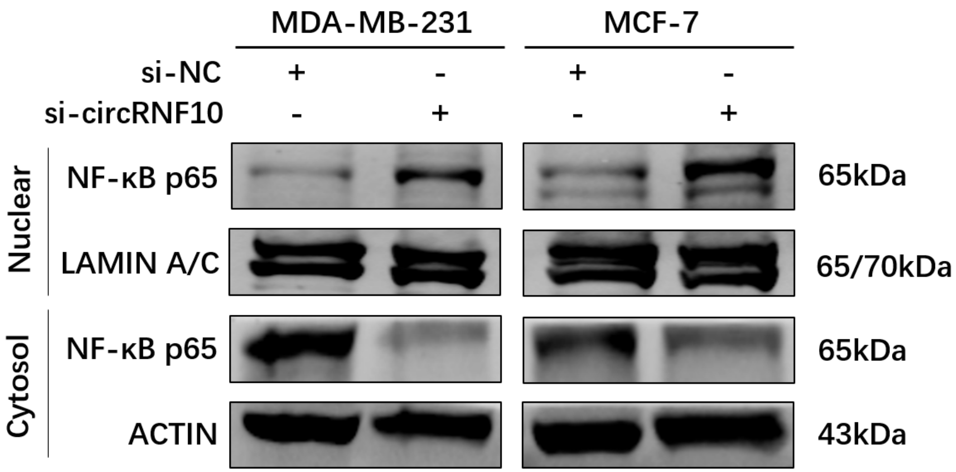
MDA-MB-231
MCF-7
si-NC + - + -
si-circRNF10 - + - +
Nuclear
NF-κB p65
LAMIN A/C
Cytosol
NF-κB p65
ACTIN
65kDa
65/70kDa
65kDa
43kDa

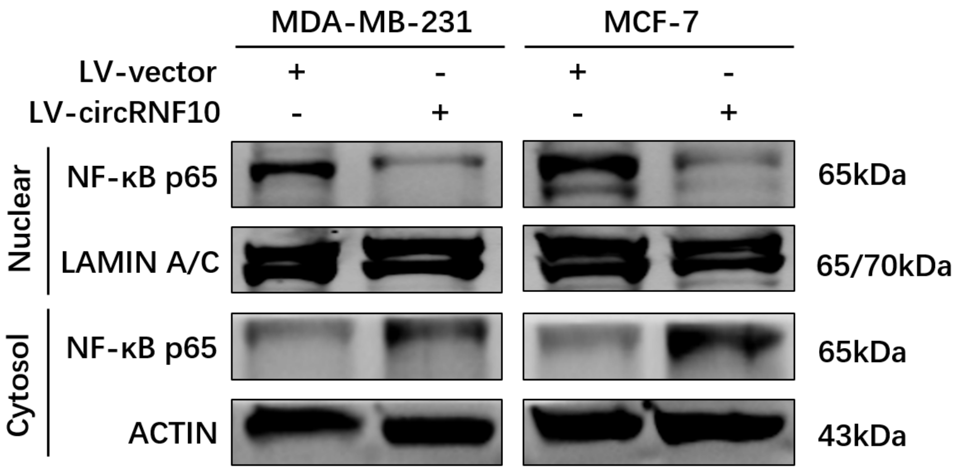
MDA-MB-231
MCF-7
LV-vector + - + -
LV-circRNF10 - + - +
Nuclear
NF-κB p65
LAMIN A/C
Cytosol
NF-κB p65
ACTIN
65kDa
65/70kDa
65kDa
43kDa

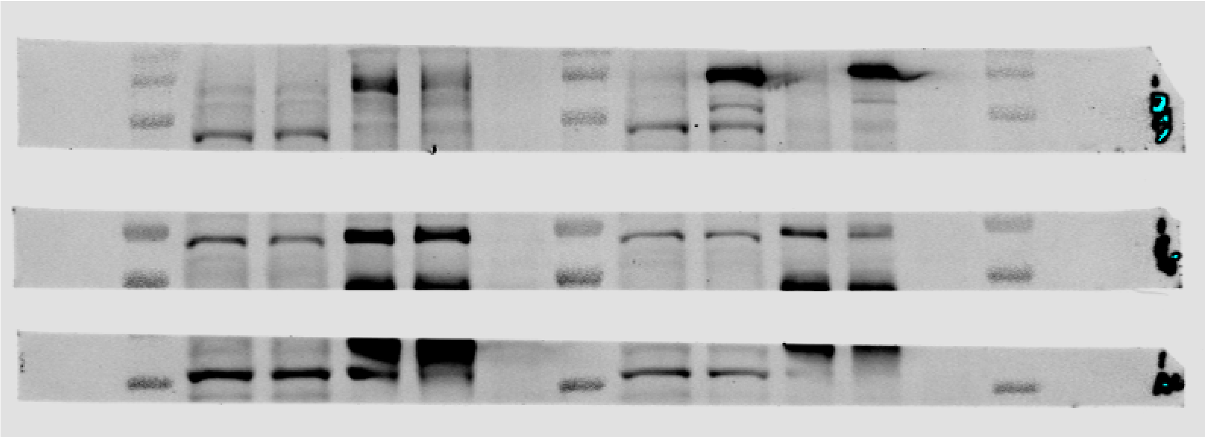

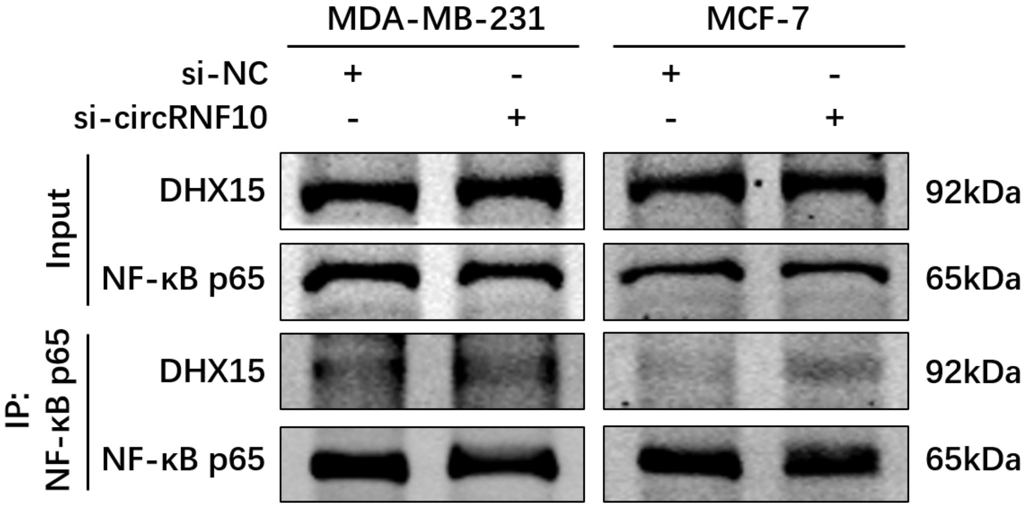
MDA-MB-231
MCF-7
si-NC + - + -
si-circRNF10 - + - +
Input
DHX15
NF-κB p65
IP:
NF-κB p65
DHX15
NF-κB p65
92kDa
65kDa
92kDa
65kDa

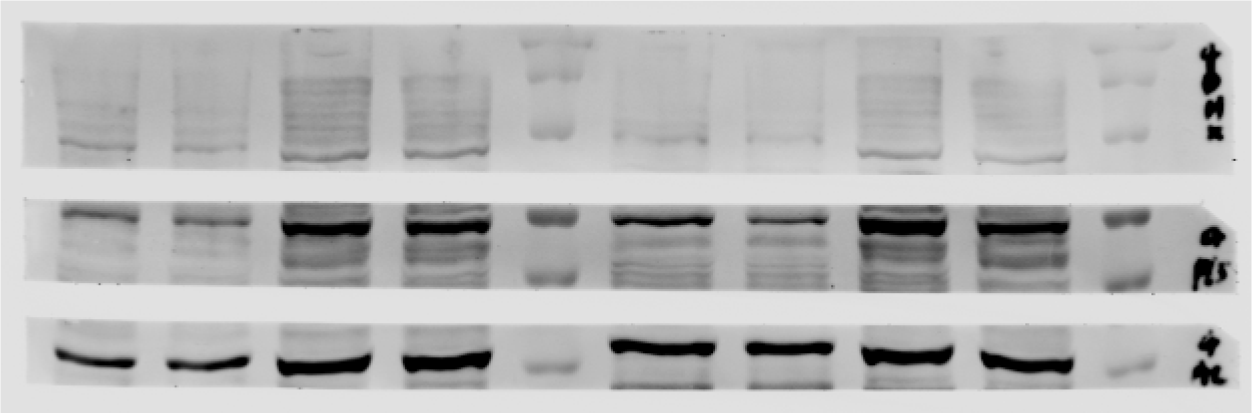

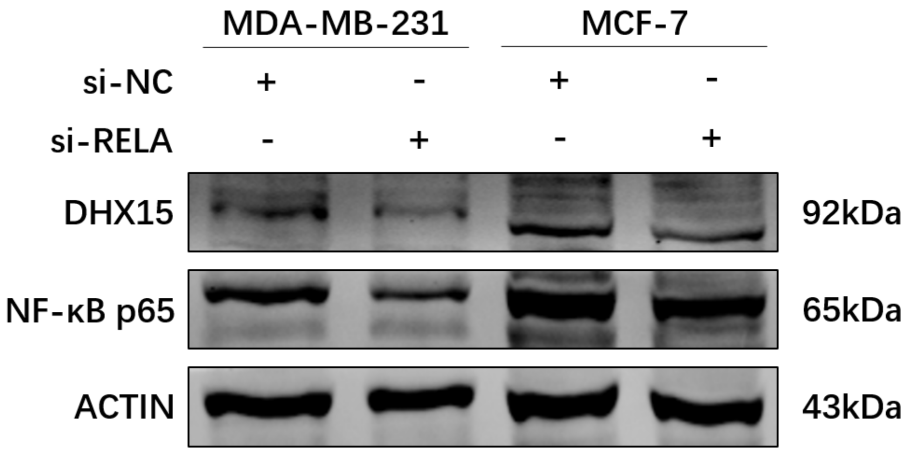
MDA-MB-231
MCF-7
si-NC
+
-
+
-
si-RELA
-
+
-
+
DHX15
92kDa
NF-κB p65
65kDa
ACTIN
43kDa

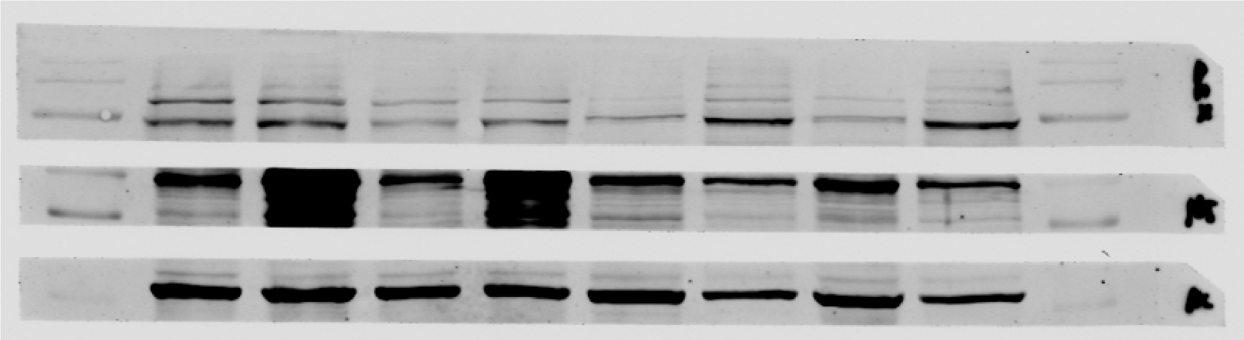

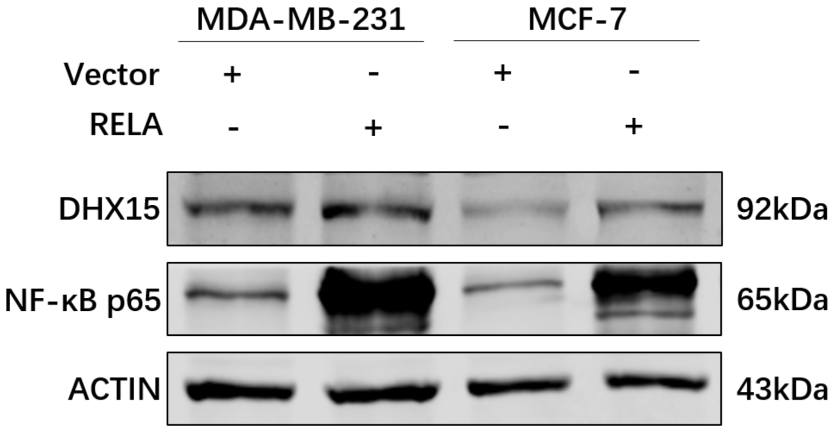
MDA-MB-231
MCF-7
Vector + - + -
RELA - + - +
DHX15
92kDa
NF-κB p65
65kDa
ACTIN
43kDa

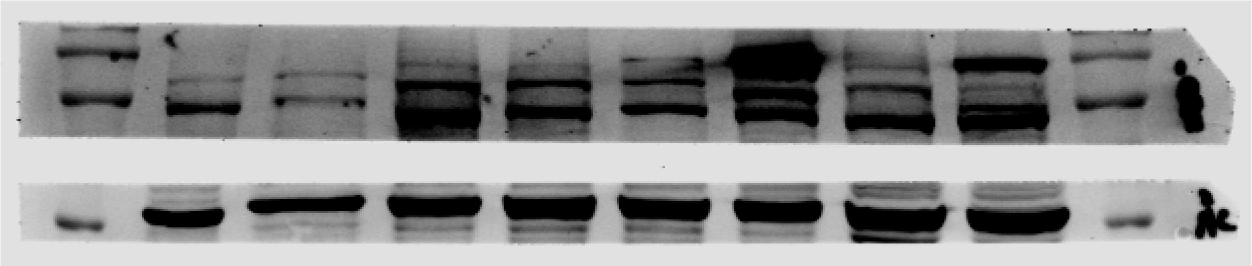

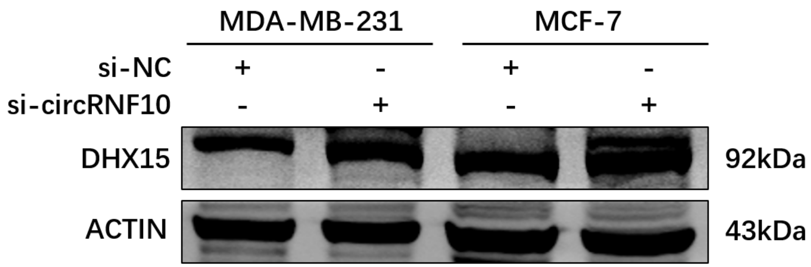
MDA-MB-231
MCF-7
si-NC + - + -
si-circRNF10 - + - +
DHX15
92kDa
ACTIN
43kDa

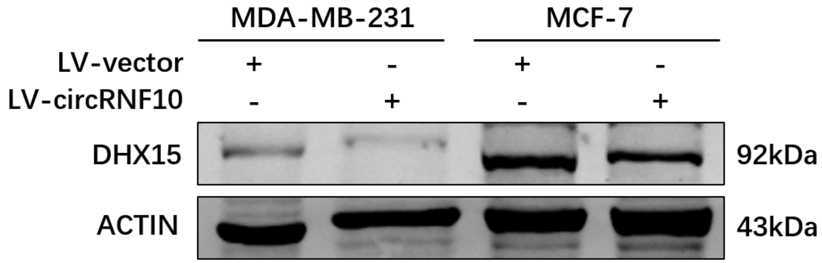
MDA-MB-231
MCF-7
LV-vector + - + -
LV-circRNF10 - + - +
DHX15
92kDa
ACTIN
43kDa
